# PIEZO1-HaloTag hiPSCs: Bridging Molecular, Cellular and Tissue Imaging

**DOI:** 10.1101/2023.12.22.573117

**Published:** 2023-12-23

**Authors:** Gabriella A. Bertaccini, Elizabeth L. Evans, Jamison L. Nourse, George D. Dickinson, Gaoxiang Liu, Ignasi Casanellas, Sayan Seal, Alan T. Ly, Jesse R. Holt, Shijun Yan, Elliot E. Hui, Mitradas M. Panicker, Srigokul Upadhyayula, Ian Parker, Medha M. Pathak

**Affiliations:** 1 Department of Physiology and Biophysics, University of California, Irvine, Irvine, CA.; 2 Sue and Bill Gross Stem Cell Research Center, University of California, Irvine, Irvine, CA.; 3 Center for Complex Biological Systems, University of California, Irvine, Irvine, CA.; 4 Department of Biomedical Engineering, University of California, Irvine, Irvine, CA.; 5 Department of Neurobiology and Behavior, University of California, Irvine, Irvine, CA; 6 Advanced Bioimaging Center, Department of Molecular and Cell Biology, University of California, Berkeley, United States; 7 Molecular Biophysics and Integrated Bioimaging Division, Lawrence Berkeley National Laboratory, Berkeley, United States; 8 Chan Zuckerberg Biohub, San Francisco, United States

## Abstract

PIEZO1 channels play a critical role in numerous physiological processes by transducing diverse mechanical stimuli into electrical and chemical signals. Recent studies underscore the importance of endogenous PIEZO1 activity and localization in regulating mechanotransduction. To enable physiologically and clinically relevant human-based studies, we genetically engineered human induced pluripotent stem cells (hiPSCs) to express a HaloTag fused to endogenous PIEZO1. Combined with super-resolution imaging, our chemogenetic approach allows precise visualization of PIEZO1 in various cell types. Further, the PIEZO1-HaloTag hiPSC technology allows non-invasive monitoring of channel activity via Ca^2+^-sensitive HaloTag ligands, with temporal resolution approaching that of patch clamp electrophysiology. Using lightsheet imaging of hiPSC-derived neural organoids, we also achieve molecular scale PIEZO1 imaging in three-dimensional tissue samples. Our advances offer a novel platform for studying PIEZO1 mechanotransduction in human cells and tissues, with potential for elucidating disease mechanisms and development of targeted therapeutics.

## Introduction

PIEZO channels are pivotal in transducing mechanical stimuli into electrical and chemical signals, and play a significant role in a wide range of physiological functions^1,2^. PIEZO1, in particular, is expressed across various excitable and non-excitable tissues, shaping key biological processes such as vascular development^3,4^, exercise physiology^5^, blood pressure regulation^6–8^, red blood cell volume regulation^9,10^, neural stem cell differentiation^11,12^, and wound healing^13,14^. The channel has been linked to several human diseases, including hereditary xerocytosis^15–18^, lymphatic dysplasia^19,20^, iron overload^21^, and malaria^22^, and ongoing studies on this recently-identified protein are likely to uncover more disease associations.

Traditionally, the study of PIEZO1 channel function has centered around patch clamp electrophysiology^1,23–25^ and, more recently, measurements of Ca^2+^ influx through the channel^11,26–30^. These methodologies have provided valuable insights into the biophysical properties of PIEZO1. However, modulation of PIEZO1 function is complex, and the nature of PIEZO1 activity, as well as its downstream outcomes, are highly context-dependent^31^. Thus, to fully understand how PIEZO1 orchestrates its diverse physiological roles and how its malfunction leads to diseases, it is crucial to study endogenous PIEZO1 in its native cellular environment. An emerging theme in PIEZO1’s physiological roles is its increased localization and activity at specific cellular structures, including focal adhesions, nuclei, and cell-cell junctions^30,32–36^, suggesting a subcellular spatial organization of PIEZO1-mediated Ca^2+^ signals. Notably, the dynamic spatial positioning of PIEZO1 in migrating keratinocytes determines their wound-healing capabilities^13^, implicating the importance of PIEZO1 spatiotemporal dynamics in governing physiological processes. Taken together, these findings highlight the need for new methodologies to precisely and non-invasively monitor the spatiotemporal organization and activity of endogenous PIEZO1.

Current methods for visualizing PIEZO1 localization predominantly utilize the fusion of fluorescent proteins such as GFP or tdTomato, but these methods are hampered by dimness, photobleaching, and an inability to measure channel activity. For measuring PIEZO1 activity, patch clamp electrophysiology is the standard assay, but it disrupts cellular mechanics and offers limited insights into the channel’s spatial localization. In contrast, PIEZO1 activity measurements using cytosolic Ca^2+^-sensitive indicators in native cells, though spatially informative, lack specificity and can be confounded by the action of other Ca^2+^-permeable channels. In addition, non-human model organisms used in many studies examining physiological roles of PIEZO1 may not fully recapitulate channel behavior in human physiology.

Here we present a novel platform to overcome these challenges and to advance physiologically and clinically relevant research on human PIEZO1. Utilizing CRISPR engineering, we introduced a self-labeling HaloTag domain fused to endogenous PIEZO1 in human induced pluripotent stem cells (hiPSCs), which can be differentiated into a variety of specialized cells and tissue organoids. Combined with the use of bright and photostable Janelia Fluor (JF)-based HaloTag ligands^37–39^, super-resolution imaging, and automated image analysis, our approach allows the study of endogenous human PIEZO1 through microscopy assays in various hiPSC-derived cell types and *in vitro* tissue organoids. This advance not only enables quantitative imaging of PIEZO1 channel localization and activity across diverse physiological scenarios but also lays the groundwork for human disease modeling of PIEZO1, large-format drug screening across various specialized cell types, and the development of targeted therapeutic interventions.

## Results

### Development and validation of PIEZO1-HaloTag hiPSC lines

**Figure 1.**
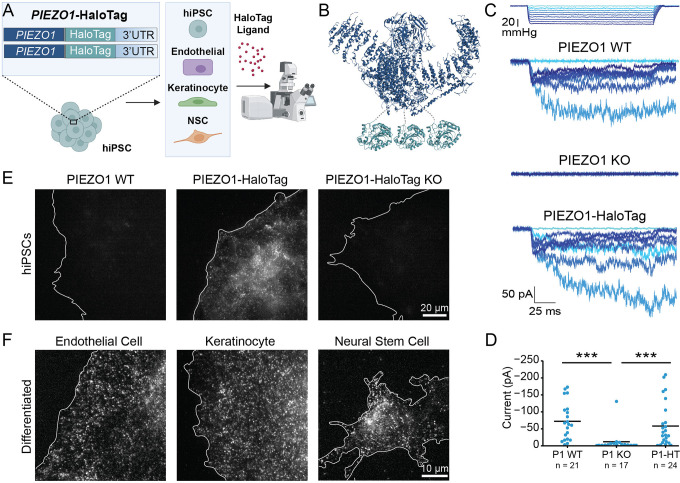
Generation and validation of the PIEZO1-HaloTag hiPSC line. **A.** Flowchart illustrating PIEZO1-HaloTag CRISPR knock-in in WTC-11 hiPSCs; multiple human cell types differentiated from the PIEZO1-HaloTag hiPSC line; and subsequent HaloTag-ligand probe labeling and imaging. **B.** Structural schematic of the trimeric PIEZO1 channel (PDB: 5Z10)^65^ with HaloTag (PDB: 5UY1)^64^ attached to the cytosolic C-terminus. Dashed grey lines highlight the linker sequence (G-S-G-A-G-A) between PIEZO1 and HaloTag. **C.** Representative traces of cell-attached patch clamp measurements with mechanical stimulation imparted through negative suction pulses for endothelial cells derived from WTC-11, PIEZO1 KO, and PIEZO1-HaloTag hiPSCs. **D.** Maximal suction-evoked current amplitudes recorded in each condition from 5 independent experiments. All values are expressed as mean ± SEM (WTC-11 mean: −71 ± 12.1 pA, n = 21; PIEZO1 KO mean: −10.6 ± 7.5 pA, n = 17; PIEZO1-HaloTag mean: −60.8 ± 13.4 pA, n =24) (*** p-value < 0.005). Cohen’s d effect sizes are −1.31 for PIEZO1 KO and −0.17 for PIEZO1-HaloTag as compared to WT. **E.** TIRF images, representative of 3 independent experiments, of a wild-type WTC-11 hiPSC without the Halo Tag introduced (left); PIEZO1-HaloTag hiPSC labeled with JF646 HTL (middle); and a PIEZO1-HaloTag Knockout hiPSC (right) showing lack of punctate labeling with the HaloTag ligand. **F.** TIRF images, representative of 3 independent experiments, of differentiated PIEZO1-HaloTag endothelial cell, keratinocyte, and neural stem cell labeled with JF646 HTL.See also Supplemental Figs. 1, 2, and 3 and Supplemental Videos 1, 2, and 3.

To create a novel, multifaceted tool for visualizing endogenous PIEZO1, we utilized CRISPR engineering to tag the endogenous channel with HaloTag, a modified bacterial haloalkane dehydrogenase that covalently binds to exogenously-provided chloroalkane HaloTag ligands^40^. We performed the genetic edits at both copies of *PIEZO1* in hiPSCs, which are capable of self-renewal as well as differentiation into a variety of specialized cell types and tissue organoids. We attached the HaloTag to the C-terminus of PIEZO1, as this location has previously been used for endogenous PIEZO1 tagging without affecting channel function^3^. Thus, PIEZO1-HaloTag hiPSCs, as well as their differentiated progeny, expressed HaloTag protein fused to endogenous PIEZO1, enabling covalent labeling with cognate HaloTag ligands (Fig. 1A, B).

We initially confirmed the effective generation of the PIEZO1-HaloTag fusion protein by western blot (Supplemental Fig. 1). Using an anti-PIEZO1 antibody, we observed a band in whole-cell lysate from the parent WTC-11 hiPSCs at the expected size of approximately 289 kDa for PIEZO1 and from PIEZO1-HaloTag hiPSCs at approximately 319 kDa. This increase in mass is consistent with fusion of HaloTag (33 kDa) to PIEZO1. As an orthogonal assay, we used an anti-HaloTag antibody and observed a signal in the PIEZO1-HaloTag hiPSC line, also at approximately 319 kDa, matching the mass observed with the anti-PIEZO1 antibody. The protein band at 319 kDA was absent in the parent WTC-11 line. To further confirm HaloTag incorporation to PIEZO1, we knocked out *PIEZO1* in the engineered PIEZO1-HaloTag hiPSCs. The PIEZO1-HaloTag Knockout hiPSCs did not exhibit a band using either the anti-PIEZO1 or the anti-HaloTag antibody. Taken together, we conclude that the heavier band corresponding to 319 kDa represents the endogenous PIEZO1 channel fused to the HaloTag domain and that the genetic modification results in the tagging of all PIEZO1 proteins produced by the cell.

To determine whether the fusion of the HaloTag to PIEZO1 affected the channel’s function, we evaluated PIEZO1 ionic currents by cell-attached patch clamp electrophysiology using hiPSC-derived endothelial cells, which show high expression of PIEZO1^3,4^. Cell-attached patch clamp measurements, with mechanical stimulation of the membrane patch through negative pressure pulses, revealed mechanically-evoked currents with slow inactivation and deactivation as previously demonstrated in primary endothelial cells^41^. These currents were absent in endothelial cells differentiated from *PIEZO1*-Knockout WTC-11 hiPSCs, confirming that they represent ionic current through PIEZO1. Notably, maximal currents from endothelial cells differentiated from the PIEZO1-HaloTag hiPSCs were similar in magnitude and indistinguishable to those from endothelial cells generated from the parent hiPSC line (Fig. 1C). Together, our results indicate that the HaloTag fusion did not abrogate channel activation (Fig. 1D).

To test labeling specificity, we incubated hiPSCs with the Janelia Fluor 646 HaloTag ligand (JF646 HTL, see Methods) and imaged the cells with Total Internal Reflection Fluorescence (TIRF) microscopy (Fig. 1E). The PIEZO1-HaloTag hiPSCs displayed a punctate signal, as previously observed for endogenous tagged PIEZO1-tdTomato channels^30^. In contrast, PIEZO1-HaloTag Knockout hiPSCs and wild-type WTC-11 hiPSCs both showed little or no punctate staining. To confirm the specific labeling of PIEZO1-HaloTag channels in the differentiated progeny of PIEZO1-HaloTag hiPSCs, we differentiated PIEZO1-HaloTag hiPSCs into three cell types which are known to express PIEZO1: endothelial cells^42^, keratinocytes^13^, and neural stem cells^30^ (Fig. 1F, Supplemental Fig. 2). Upon labeling with the JF646 HTL, each differentiated cell type also exhibited a punctate signal (Fig. 1F), whereas cells differentiated from PIEZO1-HaloTag Knockout hiPSCs lacked these punctate signals (Supplemental Fig. 3).

Overall, we validated the PIEZO1-HaloTag hiPSC line as a method to attach HaloTag ligands specifically to human PIEZO1 in multiple cell types while preserving PIEZO1 function.

### PIEZO1-HaloTag imaging with high signal-to-noise and reduced photobleaching

**Figure 2.**
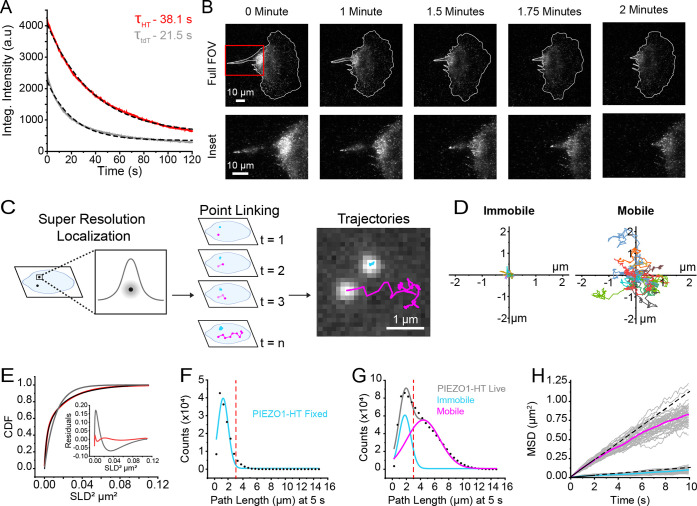
PIEZO1-HaloTag localization and tracking reveal populations of PIEZO1 with different motilities. **A.** PIEZO1-HaloTag puncta are brighter and bleach slower than PIEZO1-tdTomato puncta. TIRF image series of PIEZO1-HaloTag and PIEZO1-tdTomato endothelial cells were acquired for 2 minutes with identical acquisition settings (see Methods). Colored traces indicate the average integrated intensity of PIEZO1-HaloTag (Red; n videos = 19 from 3 independent experiments) and PIEZO1-tdTomato (Grey; n videos = 20 from 3 independent experiments) signal over time is normalized to the number of puncta in the first frame. Black dashed curves represent an exponential fit to the data with *τ*_HT_ = 38.05 s and *τ*_tdT_ = 21.48 s. **B.** A migrating PIEZO1-HaloTag NSC imaged for 2 minutes with a frame rate of 10 fps. The cell is labeled with JF646 HTL. Top row shows the entire cell (Full FOV) and the bottom row shows a zoom in of the inset box (red) on the rear of the cell. Note the channel enrichment at the rear of the cell throughout the 2-minute recording. **C.** Schematic depicting super-resolution localization, point linking, and tracking of PIEZO1-HaloTag channels labeled with JF646 HTL. Examples of two observed puncta motility behaviors: mobile (magenta) and immobile (cyan). **D.** Representative trajectories from 11 immobile and 11 mobile puncta. Starting positions of trajectories are normalized to the origin, with different trajectories indicated by color. **E.** Cumulative distribution functions (CDF) of Single Lag Displacements (SLD). Black dotted curve shows experimental data. Grey curve is a single-component exponential fit. Red curve is a two-component exponential fit. Residuals for single-component (grey) and two-component (red) fits are shown in the inset plot. **F.** Distribution of trajectory path lengths at 5 s derived from PIEZO1-HaloTag endothelial cells labeled with JF646 HTL imaged after fixing (number of tracks = 14,943 from 13 files). Dashed red line indicates cutoff location to define immobile trajectories. **G.** Corresponding distribution of trajectory path lengths for tracks at 5 s derived from live PIEZO1-HaloTag endothelial cells labeled with JF646 HTL (number of tracks = 889,971 from 40 videos). Grey curve represents a fit to a sum of two Gaussian curves; the individual Gaussian curves are shown in cyan and magenta. Dashed red line indicates cutoff location to separate mobile and immobile trajectories **H.** Mean squared displacement (MSD) of immobile (cyan) and mobile puncta (magenta). Tracks that had a path length > 3 μm were characterized as mobile (magenta) and tracks which had a path length < 3 μm were characterized as immobile (cyan) in the Gaussian curve. Each gray line represents mean MSD for all mobile puncta in a video (upper traces) and for all immobile puncta in a video (lower traces); data from 39 videos are plotted. Solid magenta (mobile) and cyan (immobile) curves are mean MSD curves across all videos. Solid black line is a linear fit to the initial MSD t < 2s, dashed black line beyond 2s represents extrapolation line. All values are expressed as mean ± SEM. Data for panels D - H are from 4 independent experiments. See also Supplemental Video 4.

PIEZO1 has been shown to be mobile in the plasma membrane^30,32,42,43^ and we confirmed this in hiPSC-derived neural stem cells, keratinocytes, and endothelial cells differentiated from PIEZO1-HaloTag hiPSCs and labeled with JF646 HTL (Supplemental Videos 1, 2, and 3). Studies on the mobility of the endogenous channel have utilized cells harvested from a PIEZO1-tdTomato knock-in mouse^30,42^ where the tdTomato fluorescent protein is limited by rapid photobleaching and low signal-to-noise ratio. We compared the spatial and temporal resolution of data obtained from PIEZO1-tdTomato and PIEZO1-HaloTag endothelial cells (Fig. 2A). Several HaloTag ligands utilize Janelia Fluor fluorophores^37^ which possess improved brightness and photostability compared to current fluorescent proteins. We compared the brightness of PIEZO1-HaloTag puncta in endothelial cells with that of puncta in endothelial cells harvested from the PIEZO1-tdTomato reporter mouse. For this, we labeled PIEZO1-HaloTag endothelial cells with Janelia Fluor 549 HaloTag ligand (JF549 HTL), enabling consistent experimental settings, i.e. the same 561 nm laser wavelength and power settings, filters, and camera acquisition settings as for tdTomato. The initial intensity of JF549 HTL-labeled PIEZO1-HaloTag puncta (4179 a.u.) was approximately twice that of the PIEZO1-tdTomato puncta (2420 a.u.), and they bleached more slowly, (time constant τ = 38.1 s for PIEZO1-HaloTag vs. τ = 21.5 s for PIEZO1-tdTomato puncta) (Fig. 2A). The mean signal-to-background ratio was 5.62 ± 0.19 for 99 PIEZO1-HaloTag puncta as compared to 3.14 ± 0.12 for 104 PIEZO1-tdTomato puncta (here and throughout all means shown as ± SEM). Thus, PIEZO1-HaloTag puncta were brighter and bleached more slowly than PIEZO1-tdTomato channels.

To illustrate the improved imaging quality of the PIEZO1-HaloTag system, we monitored by TIRF microscopy a migrating PIEZO1-HaloTag neural stem cell labeled with JF646 HTL (Fig. 2B, Supplemental Video 4). We previously showed that PIEZO1 is enriched at the rear of migratory cells; however, rapid photobleaching of the PIEZO1-tdTomato fluorophore precluded fast imaging of PIEZO1 dynamics during cell migration^13^. By imaging PIEZO1-HaloTag channels in a migrating neural stem cell over 2 minutes at a temporal resolution of 10 frames per second (fps) (Fig. 2B, Supplemental Video 4), we observed PIEZO1 channel enrichment at the trailing end of the cell throughout rear retraction. A subset of PIEZO1 puncta appeared to be organized in and moving along distinct linear arrays. The faster imaging afforded by the PIEZO1-HaloTag approach opens up avenues for mechanistic studies on PIEZO1 localization dynamics during cell migration and other physiological processes.

### Super-resolution tracking of PIEZO1 puncta reveals distinct mobility modes

To analyze the mobility of PIEZO1 puncta in the membrane, we used the FIJI plugin ThunderSTORM^44,45^ to carry out super-resolution localization of PIEZO1-HaloTag puncta in TIRF image stacks with a precision of ~20 nm. We extracted the puncta localizations and then linked successive localizations to form trajectories using the custom-built, open-source image processing software FLIKA^46^(Fig. 2C). Visual inspection of the trajectories revealed the presence of two distinct populations of puncta: those that appeared immobile and others that were highly motile (Fig. 2D), concordant with recent findings by Tyagi et al^42^.We then calculated Single Lag Displacements (SLD), i.e the distance covered by a punctum between consecutive frames, and generated a cumulative distribution function (CDF) of SLD^2^ from the individual PIEZO1 trajectories (Fig. 2E). In a population with a single diffusive behavior, the CDF follows a single exponential function. However, our data was not adequately fit by a single-component exponential; whereas a two-component exponential fit well (Fig. 2E), consistent with the presence of two distinct motility behaviors.

To segregate the two populations we separated the tracks of individual puncta based on the total path length traveled within a period of 5 seconds. We first determined how much apparent movement would result from localization error by labeling PIEZO1-HaloTag endothelial cells with JF646 HTL and imaging them after fixing the samples. The apparent path lengths over 5s for these fixed puncta followed a roughly Gaussian distribution peaking at about 1.20 μm (Fig. 2F). In contrast, trajectories from live PIEZO1-HaloTag endothelial cells labeled with the same probe showed a distribution of path lengths that was fitted well by a two-component Gaussian distribution (Fig. 2G). The first component of the Gaussian fit (peak 1.78 μm) approximated that of the fixed cell data, suggesting that this fraction of PIEZO1 puncta is indeed almost immotile in live cells. To extract the second, mobile component, we selected a cutoff value of path length of 3 μm or more at 5s to largely exclude ‘immotile’ puncta (see dashed red lines in Fig. 2F and Fig. 2G). Fig. 2H shows plots of the mean squared displacements (MSD) vs. time for classes of immotile and motile puncta segregated by this criterion. Traces for these populations separated into two distinct groups (Fig. 2H). Puncta undergoing Brownian (random) diffusion would display a straight line on this plot, with the diffusion coefficient D = d^2^/4t, where d is the mean distance from origin at time t. A linear fit up to 2 s for the immotile population yielded an apparent diffusion coefficient of 0.003 μm^2^/s. In contrast, the diffusion coefficient for the motile population based on the linear fit up to 2 s yielded a diffusion coefficient of 0.029 μm^2^/s. At longer times the relationship fell below linear (Fig. 2H), indicating sub-Brownian, anomalous diffusion.

### Capturing PIEZO1 activity with a Ca^2+^-sensitive HaloTag ligand

The availability of Ca^2+^-sensitive HTLs^38^ enables measurement of channel activity using the PIEZO1-HaloTag system. We labeled PIEZO1-HaloTag endothelial cells with Janelia Fluor 646-BAPTA HaloTag Ligand (JF646-BAPTA HTL), a non-ratiometric Ca^2+^-sensitive fluorescence indicator^38^. The location of the HaloTag at the C-terminus, near the cytosolic pore of the channel (Fig. 1B), optimally places the probe to monitor instantaneous Ca^2+^ flux as increases in local fluorescence intensity (“flickers”).

**Figure 3.**
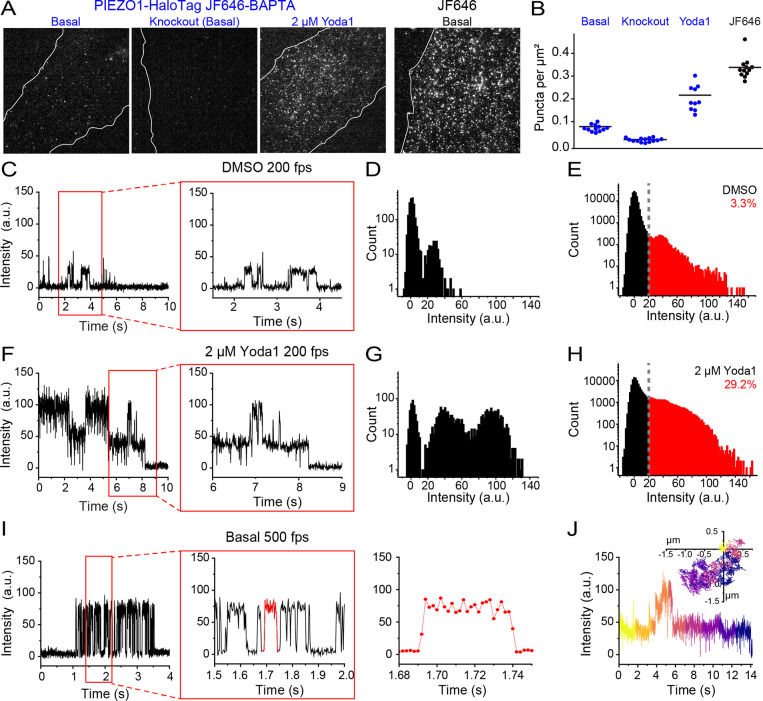
PIEZO1-HaloTag enables imaging of endogenous PIEZO1 activity with temporal resolution approaching that of patch-clamp electrophysiology. **A.** TIRF images of endothelial cells differentiated from PIEZO1-HaloTag and PIEZO1-HaloTag Knockout lines labeled with JF646-BAPTA HTL. Figure also shows PIEZO1-HaloTag endothelial cells labeled with JF646-BAPTA HTL and treated with 2 μM Yoda1. Rightmost panel shows a TIRF image of a PIEZO1-HaloTag endothelial cell treated with JF646 HTL. **B.** JF646-BAPTA HTL puncta density. All values are expressed as mean ± SEM. Basal (mean = 0.07 ± 0.004 puncta per μm^2^), PIEZO1-HaloTag KO (mean = 0.02 puncta per μm^2^ ± 0.002), 2 μM Yoda 1 (mean = 0.22 puncta per μm^2^ ± 0.02), and JF646 HTL (mean = 0.34 puncta per μm^2^ ± 0.01). Data are from 4 independent experiments. All groups were significantly different from one another (*** p-value Mann-Whitney < 0.005 for all conditions). Cohen’s d effect sizes of PIEZO1-HaloTag treated with 2 μM Yoda1 compared to PIEZO1-HaloTag (2.28) and of PIEZO1-HaloTag Knockout compared to PIEZO1-HaloTag (−4.46) **C.** Representative background-subtracted fluorescence intensity trace of an immobile PIEZO1-HaloTag punctum from TIRF imaging of a hiPSC-derived endothelial cell labeled with the Ca^2+^-sensitive HaloTag Ligand JF646-BAPTA HTL and treated with vehicle control, DMSO. Intensity profiles were plotted from tracked immobile puncta. *Right*, expanded trace from within the red box marked on the left. **D.** An all-points histogram of intensity levels from the 10 s trace in C. Counts are shown on a log_10_ scale. **E.** Combined histogram from 22 immotile puncta recorded from DMSO-treated PIEZO1-HaloTag endothelial cells (Supplemental Figure 5). **F.** Representative trace, as in C, of a punctum from a PIEZO1-HaloTag endothelial cell treated with 2 μM Yoda1. **G.** An all-points histogram of intensities from the 10 s trace in F. **H.** Combined histogram from 22 puncta in the presence of Yoda 1 (Supplemental Figure 6). **I.** Representative trace of a JF646-BAPTA HTL labeled PIEZO1-HaloTag punctum imaged using TIRF at a frame rate of 500 fps. Other details are the same as in panels C and F. Note the improved temporal resolution of PIEZO1-mediated Ca^2+^ signals illustrated by the progressively expanded traces on the right. **J.** Representative trajectory and fluorescence intensity profile of a mobile punctum labeled with JF 646-BAPTA. The inset Cartesian coordinate plot illustrates the trajectory with the starting position normalized to the origin, and time depicted by progressively cooler colors. The graph shows the corresponding background-subtracted fluorescence intensity trace. Data for panels D - H are from 3 independent experiments. See also Supplemental Figs. 4, 5, 6, and 7 and Supplemental Videos 5, 6, 7, and 8.

Endothelial cells differentiated from the PIEZO1-HaloTag hiPSCs incubated with JF646 BAPTA HTL and imaged in a bath solution containing 3 mM Ca^2+^ showed a relatively sparse mean density of puncta in individual video frames (0.07 puncta per μm^2^ ± 0.004 SEM) as compared to labeling with JF646 HTL (mean = 0.34 puncta per μm^2^ ± 0.01 SEM) (Fig. 3A). Endothelial cells derived from the PIEZO1-HaloTag Knockout hiPSC had a very low density of 0.02 μm^−2^ ± 0.002 SEM. To verify that detected JF646-BAPTA HTL signals reflect PIEZO1 activity, we applied Yoda1, a chemical agonist of the channel that increases the open-state occupancy of PIEZO1^28,47^. Incubating PIEZO1-HaloTag endothelial cells with 2 μM Yoda1 substantially increased the mean numbers of puncta observed (Fig. 3A and B), increasing from 0.07 μm^−2^ ± 0.004 SEM to 0.22 puncta per μm^2^ ± 0.02 SEM.

### Monitoring activity of endogenous human PIEZO1 with high temporal resolution

To visualize the channel activity dynamics, we imaged PIEZO1-HaloTag endothelial cells labeled with JF646-BAPTA HTL at a frame rate of 200 fps. For simplicity, we initially studied signals from immotile puncta (Supplemental Videos 5 and 6). These showed brief flickers of increasing fluorescence (i.e. fluorescence > 20 au), that could be clearly resolved in fluorescence traces and distribution histograms from individual puncta in untreated samples as well as in samples treated with vehicle control DMSO (Fig. 3C, D; Supplemental Videos 5 and 6; Supplemental Figs. 5 and 6). Between flickers, the signal at PIEZO1-HaloTag puncta sites in most recordings was generally indistinguishable from the background fluorescence at surrounding regions, indicating that the intrinsic fluorescence of the BAPTA probe is very low at resting cytosolic [Ca^2+^]. The absolute fluorescence values corresponding to the peaks of each flicker level varied between different puncta (Supplemental Fig. 4 and 5) - possibly a result of different axial locations of the plasma membrane within the evanescent field - so that a cumulative intensity distribution from 22 puncta from 16 cells showed only a single, broad component in excess of the baseline level (Fig. 3E). Treatment with Yoda1 increased the proportion of time that puncta exhibited increased fluorescence, concordant with the action of this agonist to promote activation of PIEZO1 (Fig. 3F, G; Supplemental Video 7; Supplemental Fig. 6). A cumulative intensity distribution of 22 puncta from 13 cells in the presence of Yoda1 (Fig. 3H) showed that the occupancy in the active state (fluorescence > 20 au) increased to 29.2%, as compared to 3.3% in the presence of DMSO (Fig. 3E) and untreated control cells (4.9%; Supplemental Fig. 7).

To further evaluate the temporal resolution of our recordings, we imaged JF646-BAPTA HTL signals in endothelial cells at a faster frame rate of 500 fps (Fig. 3I). Transitions on both the rising and falling phases of flickers were complete within 2 frames (Fig. 3I), indicating that the fluorescence recordings track channel gating with a time resolution of 4 ms or better.

Finally, we extended our analysis to mobile JF646-BAPTA HTL-labeled puncta, achieving simultaneous detection of channel location and activity (Fig. 3J, Supplemental Video 8). However, a current limitation is that the dim basal fluorescence of the JF646-BAPTA probe generally precluded the detection of a punctum when it was inactive, so tracks were restricted to durations when a punctum was in the bright state or could be interpolated across dim state gaps of a few frames. Taken together with measurements from immobile puncta above, this demonstrates that both immobile and mobile PIEZO1 puncta can be active.

### Imaging PIEZO1 in a Tissue Organoid Model of Neural Development

**Figure 4.**
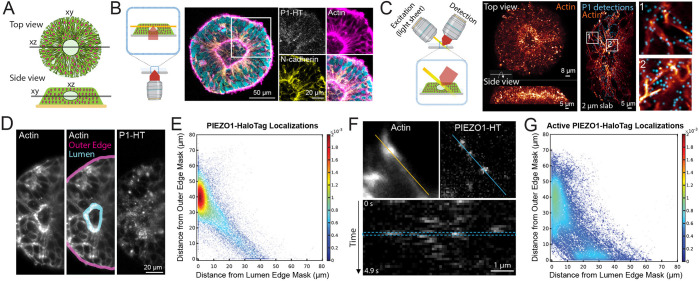
Visualizing the spatial distribution of PIEZO1-HaloTag puncta in micropatterned neural rosettes (MNRs). **A.** Top and side view schematics of an MNR, illustrating cells organized radially around a central lumen. **B.** Schematic view on the left shows the confocal microscopy imaging plane parallel to the coverslip (in orange) in an MNR. Images show a representative confocal slice of a PIEZO1-HaloTag MNR labeled with JF646, fixed, and then stained with phalloidin (magenta), anti-N-cadherin antibody (yellow) and Hoechst (cyan). The section within the white square is shown zoomed in to the right for each channel. A gamma of 0.5 was applied on the zoomed-in actin image. Note the actin-rich regions at the lumen and outer edges and the PIEZO1-HaloTag localization at the cell-cell interfaces. **C.** Schematic view on the left shows the orientation of the lattice light-sheet microscopy imaging plane in MNRs. Images on left show representative volumetric rendering of an actin-labeled MNR showing top-down and side views. The middle image panel shows a representative 2-μm slab projection of actin (orange) with PIEZO1 detections (cyan) and two zoomed insets at far right. **D.** Representative maximum intensity projection (MIP) image of 3-plane stacks of 30 frames acquired 215 nm apart of an MNR labeled with actin (*left)*, same image with the lumen and outer edge masks drawn for analysis (*middle)*, and PIEZO1-HaloTag labeled with JF635 HTL (*right*). **E.** Density scatter plot of distances of JF635 HTL labeled puncta localizations to the lumen edge mask (X axis) and to the outer edge mask (Y axis) of the MNR (n = 103 videos from 21 MNRs). The color scale indicates the relative density of puncta at each position in the scatter plot, normalized to the total number of puncta represented in the plot. Note the enrichment of PIEZO1 channels near the lumen edge mask (red cluster). Density scatter plots for each individual MNR sample can be found in Supplemental Fig. 8. **F.** Top: Representative MIPs from 3-plane stacks of actin and of Ca^2+^-sensitive JF646-BAPTA labeled PIEZO1-HaloTag puncta in an MNR. Note how puncta along the blue line in the right panel localize with actin along the orange line in the left panel. The blue line in the PIEZO1-HaloTag panel indicates the region of interest used to generate the kymograph at the bottom. Note the flickering behavior of BAPTA-labeled puncta, indicating fluctuations in channel activity levels. **G.** Same as E but for JF646-BAPTA HTL labeled active PIEZO1-HaloTag puncta (n = 39 videos from 12 MNRs). Active channels localize primarily near the actin-rich lumen, with a smaller cluster near the actin-rich outer edge of the MNR. Density scatter plots for each individual JF646-BAPTA HTL sample can be found in Supplemental Fig. 11. See also Supplemental Figs. 9, 10, 12, and 13 and Supplemental Video 9.

The PIEZO1-HaloTag hiPSC line enables the investigation of PIEZO1 at the tissue scale using organoid models derived from hiPSCs. We assessed PIEZO1 localization and activity in Micropatterned Neural Rosettes (MNRs)^48^,^49^, an hiPSC-derived human organoid model that mimics early neural development. MNRs exhibit a reproducible radial cell organization around a single central lumen, analogous to the cross-section of a neural tube (Fig. 4A). We generated MNRs from PIEZO1-HaloTag hiPSCs, labeled them with JF646 HTL, fixed the samples, and imaged them using confocal microscopy. This revealed actin-rich lumen and outer edges with actin radiating outwards from the lumen edge, and punctate HTL signal at cell-cell interfaces, which are more pronounced closer to the central lumen than at the MNR’s outer edge (Fig. 4B).

Due to the limitations of confocal microscopy for volumetric time series imaging, we employed adaptive optical lattice light-sheet microscopy (AO-LLSM)^50^ for imaging live MNRs. This system allowed for rapid, high sensitivity 3D imaging with minimal phototoxicity. We labeled live PIEZO1-HaloTag MNRs with JF635 HTL, as well as markers for actin and nuclei to delineate MNR morphology. We computationally separated the punctate PIEZO1 signal from diffuse, non-specific autofluorescence also observed in PIEZO1-HaloTag Knockout MNRs and unlabeled PIEZO1-HaloTag MNRs (see Methods and Supplemental Results). AO-LLSM imaging revealed the central lumen, the radiating actin cytoskeleton and the PIEZO1-HaloTag JF635 HTL signal throughout the MNR volume (Fig. 4C, Supplemental Video 9).

For quantitative analysis of PIEZO1-HaloTag puncta distribution in live MNRs, we captured 3D time series for an average duration of 5.5 seconds, and generated a maximum intensity projection image (MIP) of the 3 planes. We manually outlined masks representing the lumen and outer edges of the MNR in the MIP images (Fig. 4D) and computationally identified PIEZO1-HaloTag puncta, measuring their distances from these masks (see Methods). Density scatter plots showed significant clustering of PIEZO1-HaloTag puncta near the lumen edge mask (Fig. 4E, Supp. Fig. 8). PIEZO1-HaloTag puncta distances are distributed with a mode close to 0 μm from the lumen edge mask and ~40 μm from the outer edge mask (Supp. Fig. 9A), further supporting lumen enrichment. Cumulative distribution plots of distance data showed that 38 ± 1% of detected PIEZO1 puncta were within 5 μm of the lumen edge mask, while only 9 ± 1% were within 5 μm of the outer edge mask (Supp. Figs. 9A and 10A), indicating significant PIEZO1 concentration of PIEZO1 channels at the lumen border.

To determine the pattern of PIEZO1 channel activity in MNRs, we labeled PIEZO1-HaloTag MNRs with the Ca^2+^-sensitive JF646-BAPTA HTL. Similar to TIRF measurements, we observed puncta exhibiting flickering behavior (Fig. 4F), albeit with lower temporal resolution of the lightsheet modality. We computationally thresholded active PIEZO1-HaloTag puncta based on puncta intensity (see Methods) and then quantified the distances of active PIEZO1-HaloTag puncta from the MNR lumen and outer edge masks, as for the JF635 HTL-labeled puncta above. We noted a diffuse cluster near the lumen edge mask and an additional smaller cluster near the outer edge mask (Fig. 4G, Supp Fig. 11). The localization of active PIEZO1 channels in the vicinity of the actin-rich lumen and edge mask regions suggests that channels in these regions are activated by cell-generated actomyosin forces, as we previously demonstrated in single cells^30^. Cumulative distribution plots of distance data revealed that 28 ± 3% of detected active PIEZO1 puncta localized within 5 μm of the lumen edge mask, while 20 ± 5% were within 5 μm of the outer edge mask (Supp Figs. 9B and 10B). Therefore, there were only 40% more active channels at the lumen border than near the outer edge, even though there were over four times as many channels overall at the lumen, suggesting that tissue forces at the outer edge more efficiently activate PIEZO1 channels. These observations open future research avenues examining specific mechanisms by which channels are preferentially recruited to the lumen border, and how cell- and tissue-level forces in MNRs may regulate channel activity.

## Discussion

In this study, we genetically engineered a human induced pluripotent stem cell (hiPSC) line by fusing a HaloTag protein to endogenous PIEZO1. By editing both alleles of the PIEZO1 gene, we ensure that all expressed PIEZO1 protein is tagged with a HaloTag. This modification creates a chemogenetic tag for the channel that is compatible with a diverse array of specially designed HaloTag ligands (HTLs). We focus on imaging-based applications, utilizing the array of bright and photostable Janelia Fluor HTLs to visualize the localization and activity of individual PIEZO1 channels in a range of hiPSC-derived cell types and tissue organoids. Additional applications of the HaloTag technology, such as biochemical studies to identify interacting partners^51^, high-yield protein purification^40^, and Protac-mediated targeted protein degradation^52^ are also possible. Our PIEZO1-HaloTag hiPSC model thus synergizes the multifunctionality of HaloTag technologies with the versatility of hiPSCs, offering a significant leap over traditional methods in studying PIEZO1; a channel which has emerged as a critically important mechanotransducer in a wide range of physiological processes.

Our PIEZO1-HaloTag hiPSC line allows for the visualization of individual mechanically-activated PIEZO1 puncta in the native cellular environment. This approach circumvents the limitations of overexpression systems, such as altered channel localization and activity due to changes in channel density. Our model also offers the flexibility to differentiate into multiple cell types and tissue organoids, providing a more comprehensive understanding of PIEZO1 dynamics compared to previous studies limited to single cell types. The superior brightness and photostability of the Janelia Fluor HTLs allowed us to capture PIEZO1 localization dynamics with higher precision than previous PIEZO1-tdTomato models^13,30,42^, revealing two distinct mobility behaviors and allowing imaging of the rear-enrichment of PIEZO1 in migrating cells with greater temporal resolution than previously possible^13^. Thus, our tool paves the road for a new generation of studies to examine PIEZO1 dynamics under a variety of physiological conditions.

A key outcome of our study is the establishment of a novel, trackable, single-channel PIEZO1 activity assay within the native cellular milieu. This assay marks a significant advance over patch clamp electrophysiology, the standard method for measuring PIEZO1 activity. Whole-cell patch clamp dialyzes the cell, disrupting cellular structures and physiology, while cell-attached patch clamp imparts a large resting tension on the patch that may alter channel behavior. Neither modality provides spatial information on channel activation. In contrast, our approach maintains cell integrity, and provides highly specific labeling of endogenous PIEZO1, single-channel measurement capabilities in intact cells, simultaneous readouts of PIEZO1 mobility and activity, spatial information on PIEZO1 activation, and high spatial and temporal resolution. These significant technical advancements over previous imaging-based approaches to measure PIEZO1 activity^26–28,30,32^, demonstrate our tool to be exceptionally suited for examining both PIEZO1 localization and activity dynamics under native cellular conditions in isolated cells as well as tissue organoids. Our findings contrast with existing methodologies, such as GenEPi^53^, a genetically-encoded fluorescent reporter of PIEZO1 activity, which relies on overexpression and is limited by poor kinetics and weaker signals. In comparison, the PIEZO1-HaloTag hiPSC circumvents these pitfalls and provides a physiologically-relevant representation of PIEZO1 behavior.

Our single channel activity measurements revealed multiple levels of JF646-BAPTA HTL fluorescence intensity from single, stationary puncta. This was unexpected, as saturated binding of the probe would typically indicate three HaloTag ligands per PIEZO1 trimer and we presume each would respond almost simultaneously to Ca^2+^-flux given their proximity to the channel pore and high affinity for Ca^2+^ (K_d_ 0.14 μM^38^). We speculate therefore that two levels of fluorescence intensity in a JF646 BAPTA HTL-labeled punctum may represent a dimer of channels. Further super-resolution studies are needed to precisely determine the number of channels present within a single punctum and to draw conclusions on their mutual regulation. New generations of super-resolution techniques such as MINFLUX imaging will make such studies feasible^54,55^.

Our studies also reveal the capability of the Ca^2+^-sensitive JF646-BAPTA HTL to spatially map PIEZO1 mobility in addition to activity, and we find that both stationary and mobile puncta can be active. However, the low resting fluorescence of the HTL in the absence of bound Ca^2+^ poses challenges in measuring channel localization for closed PIEZO1-HaloTag channels. Addressing this limitation requires increased imaging sensitivity, development of HTL variants with increased resting-state brightness, and also further development of the PIEZO1-HaloTag hiPSC line with orthogonal labeling approaches.

Our PIEZO1-HaloTag hiPSC model significantly advances the study of PIEZO1 at the tissue scale through in vitro human organoid systems. Building upon prior research in mouse models that revealed PIEZO1’s role in neural systems^12^, our investigation examined its localization and activity during early neural development. For this, we employed an hiPSC-derived neural organoid model, micropatterned neural rosettes (MNRs), representing the developing neural tube. Utilizing AO-LLSM for high-resolution live imaging, we observed a notable enrichment of PIEZO1 channels at the lumen edge of MNRs. Interestingly, channels were observed to be active at both the lumen and the outer edges. These regions are characterized by high mechanical tension, indicated by prominent actin staining and previous work illustrating high traction stress in the outer edges of a micropatterned neuroectoderm model ^56^. Our observation highlights the need to consider the site-specific biology of tissues and their unique geometrical and mechanical properties when studying physiological roles of PIEZO1. The adaptability of our model, capable of differentiating into a variety of human tissue organoids, opens up opportunities for conducting biophysically and physiologically relevant studies on PIEZO1 function at the tissue scale. Furthermore, by integrating this approach with hiPSC-based models of human diseases, we establish a platform that holds significant promise for clinical and translational research. Finally, the precision in measuring activity and localization of PIEZO1 channels also opens up new avenues in drug screening for discovering novel therapeutic interventions targeting PIEZO1.

In conclusion, our study establishes a novel and powerful approach to study PIEZO1 using hiPSCs. This method enhances our understanding of PIEZO1’s role in cellular mechanics and signaling in normal physiology as well as disease conditions and holds significant potential for developing therapeutic strategies in PIEZO1-mediated human diseases. Future improvements, including continued developments in the rapidly evolving areas of HaloTag technologies as well as advanced imaging techniques, will further refine our ability to study PIEZO1’s intricate role in health and disease. More broadly, our approach could be applied to other Ca^2+^-permeable membrane proteins.

## Methods

### hiPSC Culturing and Maintenance

hiPSCs were maintained in mTeSR^™^ Plus Basal Medium (Cat. No.100–11300, STEMCELL Technologies) + Primocin (Cat. No.NC9392943, Invivogen) in an incubator at 37°C with 5% CO_2._. Cells were cultured on plates incubated with 10 μg/mL Vitronectin XF (Cat. No.07180, STEMCELL Technologies) for at least 1 hour at room temperature prior to passaging. Media was changed every other day, and cells were passaged mechanically or as single cells (10,000 cells/cm^2^) every 4–7 days. For generating single cell suspensions of hiPSCs, cells were dissociated with Accutase (Cat. No.# 07920_C, STEMCELL Technologies) for 3–5 minutes in a 37°C incubator. Accutase was diluted 1:1 with mTeSR Plus, 100ug/ml Primocin, and 10 μM Y-27632 (Cat. No.SM-0013-0010, Biological Industries, USA) and cells were centrifuged at 210 *g* for 5 minutes. For TIRF imaging, cells were seeded at 13,000 cell/cm^2^ density on Vitronectin XF coated MatTek dishes (Cat. No.P35G-1.5-14-C, MatTek Corporation Cells were regularly checked for mycoplasma contamination and karyotypic abnormalities.

### CRISPR Engineering

All PIEZO1 edits were outsourced to Sythego, Menlo Park, CA. and made in WTC-hiPSCs with normal karyotype. The ICE (Inference of CRISPR Edits) software analysis package developed by Synthego was used for analysis of CRISPR editing data. All clones had normal karyotype and passed pluripotency tests (OCT4 and SSEA immunostaining) after CRISPR editing. Karyotyping of clones was performed with Karystat analysis (ThermoFisher).

#### PIEZO1-HaloTag hiPSCs

Briefly, the strategy employed for the CRISPR engineering was as follows. hiPSCs were transfected with Cas9, sgRNA (5’-GUGGACUCGUGAGAAGGAGU-3’) and knock-in template sequence which was designed with a 5’ homology arm (HA) of 496 bp upstream of the stop codon of PIEZO1, an 18 bp linker, the HaloTag coding sequence followed by the 3’ HA containing the TAG stop codon and 499 bp of the 3’ untranslated region of PIEZO1. The 18 bp linker, ggatccggtgcaggcgcc, encodes the amino acid sequence GSGAGA. HaloTag 7 is 297 amino acids encoded by 891 bp sequence. Transfected cells were screened for the presence of the insertion by PCR of the genomic DNA with FWD primer (5’–3’): GCCAAGCTCATCTTCCTCTAC and REV primer (5’–3’): GAACATGAAGGACTTGGTGAGTA which should yield a 1,669 bp product, while unedited gDNA would yield a 760 bp product.

#### PIEZO1-HaloTag Knockout hiPSCs

PIEZO1-HaloTag hiPSC cells were edited to obtain homozygous indels in multiple exons within the PIEZO1 coding region in exons 6 and 43. Cas9 and guide RNAs were transfected sequentially into PIEZO1-HaloTag hiPSC cells. Guide RNAs used were sgRNA (5’-UGGAUGCCAGCCCGACGGCA-3’) targeting exon 6 and sgRNA (5’-UCCGCCUACCAGAUCCGCUG-3’) targeting exon 43 of Piezo1. Forward primer 5’-AGGTAGACACTGGAGAGGGC-3’ and reverse primer 5-CAGAGGAGCAGCTGTGGATG-3’ were used in PCR amplification of genomic DNA from transfected cells. Sequencing of the PCR fragment revealed a homozygous −1 indel in exon 6. Indels in exon 43 were identified using the forward primer 5’-ACCTTCTCTGTCTCTCGGCT3’ and the reverse primer 5-ACCTTCTCTGTCTCTCGGCT-3’ for PCR amplification. Sequencing of the fragment revealed a homozygous a −5 indel in exon 43 .

### hiPSC Differentiation

#### Neural Stem Cell (NSC) Differentiation

Neural stem cells were differentiated from hiPSCs using STEMdiff^™^ Neural Induction Medium (Cat. No. 05839, STEMCell Technologies) monolayer culture protocol as per the manufacturer’s instructions. Briefly, hiPSCs were passaged using Accutase as described above and resuspended in STEMdiff^™^ Neural Induction Medium containing SMADi and 10 μM Y-27632. Cells were plated at 2 × 10^5^ cells/cm^2^ onto tissue culture plates coated with 10 μg/ml of CellAdhere^™^ Laminin-521 (Cat. No.77003, STEMCELL Technologies). Media changes (without Y-27632) were performed daily and cells were passaged at day 7, 14 and 21. NSCs were used from Day 21.

#### Endothelial Cell (EC) Differentiation

Endothelial cells were differentiated following the S1-S2 method^57^. hiPSCs were dissociated into single cells using Accutase and plated on 10 μg/mL Vitronectin XF (Cat. No. 07180, STEMCELL Technologies)-coated plates with 10 μM of Y27632 (Cat. No.SM-0013-0010, Biological Industries, USA) in mTeSR^™^ Plus Basal Medium (Cat. No.100–11300) + Primocin (Cat. No.NC9392943, Invivogen). 3 × 10^5^ cells/cm^2^ cells were seeded per well of a 6-well tissue culture plate. 24 hours after seeding, the media was changed to S1 media. S1 media was prepared first by making a Basal medium consisting of Advanced Dulbecco’s modified Eagle’s medium (DMEM)/F12 (Cat. No. 12334010, Thermo Fisher Scientific ), 1x Glutamax supplement (Cat. No. 35050061, Thermo Fisher Scientific), 60 μg/ mL L-Ascorbic Acid (Cat. No. A8960, Sigma- Aldrich), and Primocin. Basal media was supplemented with 6 μM CHIR99021 (Cat. No. SML1046-5MG, Sigma-Aldrich) to create S1 media. Media was changed every 24h for two days. After two days in S1 media, media was changed to S2. S2 media was prepared by supplementing Basal media with 10μM SB431542 (Cat. No. S1067, Selleck Chem), 50 ng/mL bFGF-2 (Cat. No. 100-18B-100ug, PeproTech), 50 ng/mL VEGF-A (Cat. No. 100–20, PeproTech), and 10 ng/mL EGF (Cat. No. AF-100–15-100ug, PeproTech). Media was changed every 24h throughout the protocol. After a total of 48 hours in S2 media, cells were purified using an immunomagnetic CD31 MACs sorting kit (Cat. No. 130-091-935, Miltenyi) and 15,000 cells were plated on 10 μg/mL Fibronectin (Cat. No. 356008, Corning)-coated MatTek dishes (Cat. No. P35G-1.5-14-C, MatTek Corporation) and cultured for 48h in EGM-2 media (Cat. No.CC-3162, Lonza) before imaging.

#### Keratinocyte Differentiation

Mechanically passaged hiPSCS were plated on 10 μg/mL Vitronectin XF in 6 well TC plates in 1 μM all-trans-RA (Cat. No. R2500-25MG, Sigma-Aldrich) to NutriStem^®^ hPSC XF Medium (Growth Factor-Free)(Cat. No.06-5100-01-1A, Biological Industries Israel Beit-Haemek Ltd.). Daily media changes were performed with media containing 1 μM RA for 7 days. On day 7, differentiated cells were passaged with 2 mg/mL of Dispase (Cat. No. CnT-DNP-10, Cellntec) incubated for 30 min at 37C. Cells were resuspended in 1 mL of media CnT-Pr media (Cat. No. CNT-PR, CellnTec) and 1/10^th^ of total cells were plated on 10 μg/mL Fibronectin (Cat. No. 356008, Corning) coated MatTek dishes (Cat. No.P35G-1.5-20-C, MatTek Corporation). Cells were cultured in CnT-Prime Epithelial Proliferation Medium (Cat. No. CNT-PR, CellnTec) for 2–4 weeks prior to imaging. Media was exchanged to CnT-Prime Epithelial 2D Differentiation Medium (Cat. No. CnT-PR-D, CellnTec) for 2–4 days prior to imaging.

#### Preparation and Differentiation of Micropatterned Neural Rosettes (MNRs)

MNRS were generated from hiPSCs using a method developed by Haremaki et al. 2019^49^ using Arena A CYTOOchips (Cat. No.10-020-00-18, CYTOO INC.). A CYTOO Arena A chip was placed on parafilm in a 10-cm petri dish (Cat. No. 08757100D, Fisher Scientific) the top side was coated with 10 μg/ml of CellAdhere^™^ Laminin-521 (Cat. No.77003, STEMCELL Technologies) for 3 hours at 37°C. Laminin was then removed via multiple washes in PBS + calcium + magnesium (PBS +/+) (Cat. No. PBL02-500ML, Caisson Laboratories Inc.). The CYTOOchip was then moved to a single well of a 6 well tissue culture plate with PBS +/+ prior to plating. PIEZO1-HaloTag and PIEZO1-HaloTag Knockout hiPSCs were washed with PBS +/+ and incubated with accutase at 37°C for 3–5 minutes to generate a single cell suspension. Accutase was diluted 1:1 with mTeSR Plus, containing 10 μM Y-27632 and spun at 210 *g* for 5 minutes. Cells were resuspended, counted and seeded at a concentration of 5×10^5^ hiPSCs in 2 mL of media on top of the micropattern in a single well of a 6 well plate. The media was changed after 3 hours into differentiation media comprising of a 1:1 mixture of DMEM/F12 and Neurobasal containing 1:100 Glutamax, 1:200 Non Essential Amino Acids, 1:200 N2 supplement, 1:100 B27 without vitamin A (all Invitrogen), 3.5 μL l^−1^ 2-mercaptoethanol (Sigma), 1:4000 insulin (Sigma), 10 μM SB431542 (Cat. No. S1067, Selleck Chem) and 0.2 μM LDN193189 (Cat. No. SM-0005–0010, Biological Industries, USA). Media was changed daily and MNRs were kept in culture until day 5 in a 37°C incubator, at which time central lumens were present. MNRs were incubated with HaloTag Ligand as detailed below and imaged on day 5. To label actin structures, MNRs were incubated with SPY555-actin (Cat. No. CY-SC202, Cytoskeleton, Cytoskeleton) at 1:1000 in culture media, 1:1000 SPY505-DNA (Cat. No. CY-SC101, Cytoskeleton) was added to label DNA, both for 1 hour prior to imaging. MNRs were washed 3 times in culture media prior to imaging.

Small adjustments were made to the above protocol to prepare MNRs for confocal microscopy. The CYTOO chip was placed in the bottom of a 35 mm glass-bottom dish without coverglass (Cat. No. D35–14, Cellvis). Thus, the micropatterned surface was positioned at the bottom in the hole. Subsequently, biosafe glue (KWIK-CAST silicon sealant, World Precision Instruments) was added to the bottom of the 35 mm dish around the edges of the CYTOOchip to seal it in place. The chip was rehydrated with 2mL PBS +/+ for 5 minutes. The same protocol as above was followed, coating the chip with laminin and seeding the cells directly into the 35 mm dish. MNRs were labeled with HaloTag Ligand on Day 5 as detailed below and imaged live for AO-LLSM or after fixation for confocal imaging.

### Mouse Liver Sinusoidal Endothelial (mLSEC) Isolation and Culture

Livers from the PIEZO1-tdTomato mice^3^ were dissected and placed on a petri dish and minced with scalpel blades. Once the liver was minced, it was resuspended in a solution to further dissociate the tissue. This solution contained 1 mL 2.5 U ml-1 dispase, 9 mL 0.1% collagenase II, 1 μM MgCl_2_, and 1 μM CaCl_2_ in Hanks Buffer. The tissue was incubated with the dissociation mixture for 50 mins in a tube rotator with continuous agitation at 37°C. After the completion of the dissociation process, the dissociated tissue was filtered using 70 and 40 μm cell strainers. The dissociated cells were then washed twice in PEB buffer containing phosphate-buffered saline solution (PBS), 0.5% BSA, pH 7.2, EDTA 2 mM, and EDTA 2 mM. Once the pellets were washed, cells were placed in 1 mL PEB buffer and 30 μL CD146 microbeads (Cat. No. 130-092-007, Miltenyi Biotech) at 4°C for 15 min under continuous agitation. An LS column (Cat. No. 130-042-401, Miltenyi Biotech) was primed with PEB buffer during this incubation. Once the mLSECs were selected for using the microbeads, the solution was then placed in the primed LS column to separate the mLSECs (Miltenyi Biotech). After the cells passed through the column, the column was washed 3 times with 5 mL PEB buffer. Any CD146 positive cells were eluted from the column using 5 mL warmed EGM-2 growth medium supplemented with EGM-2 bullet kit (Lonza). The cells were pelleted with a 300 *g* spin for 5 min and counted in 1 mL EGM-2 media. Glass-bottom #1.5 dishes were prepared by coating with 10 μg/mL Fibronectin (Cat. No. 356008, Corning). After performing the cell count, approximately 30,000 cells were plated on each fibronectin coated glass-bottom dish. After 2 hours, a media change was performed and then media changed every 48 h until imaging 72 h later.

### HaloTag Ligand Treatment Protocol

After differentiation of the PIEZO1-HaloTag hiPSC lines into either NSCs, Endothelial cells, Keratinocytes, or MNRs, each respective cell type was incubated at 37 °C with 500 pM of Janelia Fluor^®^ 646 HaloTag Ligand (NSCs, ECs, or keratinocytes) (Cat. No. GA1120, Promega) or Janelia Fluor^®^ 635 HaloTag Ligand (MNRs) (Cat. No. CS315103, Promega) or Janelia Fluor^®^ 646-BAPTA-3’AM- HaloTag ligand (requested from Lavis lab, Janelia Research Campus, HHMI) for incubation time outlined in Supplementary Methods Table No.1. MNRs were treated with JF635 HTL due to its improved fluorogenic properties and compatibility with existing filter sets in the AO-LLSM system^58^. Incubations with HTLs were performed in each respective cell type’s basal media outlined in their respective culturing sections. Cells were gently washed 5 times with DMEM/ F12 1:1 Cat. No. 25116001, Invitrogen) at room temperature prior to imaging using TIRF microscopy. MNRs were washed 3 times with culture media prior to fixation or live imaging.

### Immunofluorescence

Samples plated in MatTek dishes (Cat. No. P35G-1.5-14-C or P35G-1.5-20-C, MatTek Corporation) were first fixed in a 4% v/v paraformaldehyde solution, including 5 mM MgCl_2_, 10 mM EGTA, and 40 mg/mL sucrose in PBS (pH = 7.3) for 10 minutes at room temperature. Next, the fixed cells or micropatterned neural rosettes were permeabilized with 0.3% Triton X-100 in PBS for 5 minutes. 5% BSA in PBS for 1 h at room temperature was used to block nonspecific binding of excess antibodies. After blocking was complete, the sample was incubated overnight at 4°C with primary antibody diluted in 1% BSA (Primary antibodies used and their concentrations are included in supplementary methods). Next, samples were washed several times to remove the primary antibody and then were incubated with secondary antibody for 1 hour at room temperature (Secondary antibodies used and their concentrations are included in supplementary methods). Samples were washed several times and subsequently labeled with Hoechst at 1ug/mL for 5 minutes at room temperature. Samples were stored in 1x PBS prior to imaging.

### Patch clamp

Cell-attached patch clamp experiments were made using an Axopatch 200B amplifier (Molecular Devices) at room temperature. Pipettes were made from thin walled borosilicate glass capillaries (Warner Instruments) and contained a solution consisting (in mM) of 130 NaCl,10 Hepes, 10 tetraethylammonium chloride, 8 Glucose, 5 KCl, 1 CaCl_2_, 1 MgCl_2_, (pH 7.3) with NaOH. Bath solution composition included (in mM) 140 KCl, 10 Glucose, 10 Hepes, 1 MgCl_2_, (pH 7.3 with KOH) was used to zero the membrane potential. The pipettes had a resistance of 0.6–1.1 MΩ when submerged in these solutions. Negative-suction application provided mechanical stimulation during recordings using a high-speed pressure clamp (HSPC-1; ALA Scientific) controlled using Clampex software. Suction pulses were applied using the patch pipette and membrane potential was held at −80 mV. Off-line leak subtraction was performed prior to quantification of maximum current. Maximum current was manually recorded and quantified for statistical differences using students t-test and mean effect size using Cohen’s d.

### Total Internal Reflection Fluorescence (TIRF) microscopy Imaging

TIRF microscopy was used to image endogenous PIEZO1-tdTomato and PIEZO1-HaloTag channels at 37°C. PIEZO1-HaloTag cells were incubated in accordance with the HaloTag Ligand Treatment Protocol above, and washed thrice with phenol red-free DMEM/F12 1:1 (Cat. No. 25116001, Invitrogen) and incubated in imaging solution, composed of 148 mM NaCl, 3 mM CaCl_2_, 1 mM KCl, 2 mM MgCl_2_, 8 mM Glucose, 10 mM HEPES, pH 7.30, and 316 mOsm/L osmolarity. PIEZO1-HaloTag and PIEZO1-tdTomato samples in Fig. 1, Supplemental Fig. 3, Supplemental Video 1, Supplemental Video 2, Supplemental Video 3, Fig. 2A, and Fig. 2B were all imaged using an Olympus IX83 microscope fitted with a 4-line cellTIRF illuminator, an environmental control enclosure and stage top incubator (Tokai Hit), programmable motorized stage (ASI), a PLAPO 60x oil immersion objective NA 1.45 (1 pixel is approximately 0.109 μm, no pixel binning), and a Hamamatsu Flash 4.0 v2+ scientific CMOS camera. All other samples were imaged using an Olymus IX83 microscope fitted with a 4-line cellTIRF illuminator, an environmental control enclosure and stage top incubator (Tokai Hit), programmable motorized stage (ASI), a PLAPO 60x oil immersion objective NA 1.50 (1 pixel is approximately 0.108 μm, no pixel binning), and a Hamamatsu ORCA-Fusion BT Digital CMOS camera. All images were acquired using the open-source software Micro-Manager^59^. Cells were illuminated with either a 560 nm or a 640 nm laser, as appropriate for the fluorophore used, and images were acquired with a Hamamatsu ORCA-Fusion BT Digital CMOS camera.

### Analysis of PIEZO1-HaloTag Puncta Diffusion based on TIRF imaging

To assess the diffusion properties of PIEZO1, the single-molecule localization software package ThunderSTORM^44,45^ implemented in FIJI^44,45^ was used to detect and localize single PIEZO1-HaloTag puncta observed in TIRF recordings. ThunderSTORM was set to use multi-emitter fitting. PIEZO1-HaloTag trajectories were then generated by connecting puncta localization centroids over time using the image analysis software FLIKA^46^. The nearest punctum within three pixels (centroid-centroid distance) between adjacent frames was linked and assigned to a trajectory. If no puncta were detected within adjacent frames the next frame was also searched, and if no puncta could be detected within the search radius the trajectory was terminated. Tracks with a minimum of 4-links were analyzed to calculate signal-to-background, diffusion coefficients, trajectory path length, and mean squared displacement (MSD) values. Single lag displacements (SLD) were calculated using the distance moved by the punctum between two consecutive frames. In cases where the punctum was briefly undetected (a “gap frame”), punctum positions in the gap frames were interpolated.

### Comparison of PIEZO1-tdTomato and PIEZO1-HaloTag signals

For each PIEZO1-tdTomato and PIEZO1-HaloTag JF549 HTL video, the sum of all pixel intensities in a region of interest (integrated intensity) was measured over the duration of the video in FIJI. This was done for 3 rois in each video. These values were then background-subtracted using the integrated intensity from a roi outside the cell border of the same video. ThunderSTORM was used to identify puncta in the first frame of each of the three rois and the background-subtracted integrated intensity was normalized for the number of puncta. These normalized integrated intensity values from each of the 3 rois within a video were then averaged to create a video-level average, which were then again averaged over 20 videos for PIEZO1-tdTomato and 19 videos for PIEZO1-HaloTag to yield an overall mean for each fluorophore.

To calculate the relative signal-to-background ratio for JF549-labeled PIEZO1-HaloTag and for PIEZO1-tdTomato puncta (for Fig. 2A), puncta were detected in the first frame for each video. The signal intensity was measured from a 3×3 pixel roi after subtracting camera black level. Background fluorescence was similarly determined from a 3×3 pixel roi inside the cell devoid of puncta.

### Analysis of PIEZO1-HaloTag activity based on TIRF imaging

FLIKA software was used to build trajectories from PIEZO1-HaloTag cells labeled with JF646-BAPTA and to record puncta fluorescence intensity within a 3×3 pixel roi for each frame ^46^. To account for the flickering behavior of the JF646-BAPTA HTL, the allowable gap time was 90 ms. Intensities and positions were interpolated over gap frames. Puncta intensities were background subtracted using intensities from a 3×3 pixel roi inside the cell devoid of puncta. After trajectory building, videos were visually inspected to identify representative stationary and motile puncta.

### Live MNR Imaging

Micropatterned neural rosettes (MNRs) of 140 μm in diameter grown on 19 mm square Arena A CYTOO chips (Cat. No.10-020-00-18) were imaged on a modified adaptive optical lattice light-sheet microscope (AO-LLSM)^50^. As described previously^60^, prior to all imaging, the microscope was calibrated to correct for optical aberrations from the system. The Cytoo chip was mounted on a custom designed sample holder and immersed in 40 ml of phenol-free MNR culture medium. The excitation (Thorlabs 0.6 NA, TL20X-MPL) and detection (Zeiss 1.0 NA, 421452-9800-000) objectives were also immersed into the imaging medium. The MNRs were imaged with a multiBessel square lattice light sheet with the NA_sq_ of 0.35 and a 0.3/0.4 NA annular mask. The 488 nm, 560 nm and 642 nm lasers were used to visualize SPY505-DNA (DNA, Spirochrome Cat# CY-SC101), SPY555-actin (actin, Spirochrome Cat# CY-SC202), and Janelia Fluor^®^ 635-HaloTag Ligand (Non-Bapta) or Janelia Fluor^®^ 646-BAPTA-AM- HaloTag ligand (PIEZO1), respectively, with power at the back pupil of the excitation objective of 45 μW for 488 nm, 50 μW for 560 nm and ranging between 2.1–2.9 mW for 642 nm. To balance volumetric imaging speed, signal to noise and photobleaching, images were acquired using camera exposure between 30–50 milliseconds. All data from the AO-LLSM were collected on two Hamamatsu ORCA-Fusion sCMOS cameras. Emission light from actin, DNA, and JF635 or JF646 was separated by a dichroic mirror (Chroma T600D-CRB) and passed to two cameras equipped with either Semrock FF01-600/37-25 emission filter for actin or Semrock FF01-538/685-25 filter for DNA and JF635 or JF646-BAPTA .

The 3D volumetric imaging of MNRs was performed by tiling AO-LLSM across the sample. Each tile (approximately 200 × 100 × 15 μm^3^) was scanned by moving the sample stage at 400 nm step sizes. Each neighboring tile had a 5 μm overlap between each adjacent tile. Mismatch between the excitation LLS and the detection focal plane caused by the MNR were corrected prior to every acquisition and was essential to ensure optimal imaging of PIEZO1, DNA and actin structures^61^. The AO-LLSM data was processed using MATLAB versions R2022b and R2023a. The large 3D MNR volumes were stitched in skewed space, deconvolved, deskewed and rotated on Advanced Bioimaging Center’s computing cluster at UC Berkeley using the computational pipelines published on GitHub (https://github.com/abcuc-berkeley/LLSM5DTools). The skewed space deconvolution was performed as described previously^60^ with experimentally measured point spread functions obtained from 200 nm fluorescent beads (Invitrogen FluoSpheres Carboxylate-Modified Microspheres, 505/515 nm, F8811). The nuclei were denoised using Content-Aware Image Restoration (CARE)^62^. The training data for the denoising model was collected using lattice light-sheet microscopy as previously described^60^ by volumetrically scanning LLC-PK1 cells expressing nuclear marker (H2B) to record low SNR and corresponding high SNR 3D stacks. The AO-LLSM instrument was controlled using a custom LabVIEW based image acquisition software (National Instruments, Woburn, MA) licensed from Janelia Research Campus, HHMI.

To observe the dynamics of PIEZO1-HaloTag in MNRs, 3-plane stack videos were acquired. For this, the sample stage (using the SmarAct MLS-3252 Electromagnetic Direct-Drive) was rapidly scanned across a 1 μm sample range (comprised of three image planes spaced 400 nm along the sample stage scan axis, corresponding to 215 nm along the optical z axis) at an interval between 160–210 ms per stack for 30 time points. The dynamic time series datasets were deskewed and maximum intensity projected prior to analysis^63^. The corresponding DNA and actin volumes were recorded at the same location prior to acquiring the PIEZO1-HaloTag time series. A new dataset of 30 time points was collected every ~10 μm throughout the MNR and was repeated on multiple samples.

### Detection of PIEZO1-HaloTag puncta and separation from autofluorescent and non-specific blobs

The maximum intensity projection (MIP) images of deskewed 3-plane stacks were used for puncta localization analysis. PIEZO1-HaloTag puncta localization was analyzed in JF635-labeled PIEZO1-HaloTag MNRs. JF635-labeled PIEZO1-HaloTag Knockout MNRs and unlabeled PIEZO1-HaloTag MNRs served as controls to characterize and subsequently filter out spurious detections due to autofluorescent blobs and to unbound JF635 probe, as described below.

The ImageJ plugin ThunderSTORM^44^ was used to detect PIEZO1 puncta, generating puncta localization maps for each frame of the acquisition video. A Gaussian function was fitted to the detected puncta spots to generate a centroid for each punctum, as described above for TIRF data. To distinguish labeled PIEZO1-HaloTag puncta from non-specific autofluorescent blobs, which were much larger in size and also present in PIEZO1-HaloTag KO MNRs and unlabeled PIEZO1-HaloTag MNRs, a first filtering step was applied by setting a threshold based on the standard deviation (σ) of the Gaussian fit of each punctum. Puncta with σ higher than 280 nm were removed since experimental σ measured on 642 nm beads was ~189 nm.

In order to remove any spurious detections due to unbound JF635 dye passing through the plane of imaging during acquisition, or to ThunderSTORM erroneously detecting small local background fluctuations as puncta, a time-based filtering step was applied to retain only objects that persisted over multiple frames. The custom-built FLIKA algorithm^46^ was used to link detected puncta across frames with a maximum linking distance between consecutive frames of 324 nm (3 pixels) and a linking gap of one frame, as done for TIRF data analysis above. Tracks composed of at least 3 segments (i.e. successfully linked puncta in at least 4 frames) were considered representative of bona fide PIEZO1-HaloTag puncta and retained for further analysis. See the Supplementary Results section for performance of these settings in detecting and filtering PIEZO1-HaloTag puncta.

### Detection of PIEZO1-HaloTag BAPTA signal and separation from autofluorescent and non-specific blobs

Puncta in MNRs labeled with JF646-BAPTA HTL were detected using ThunderSTORM as described above for JF635-labeled MNRs. The same size-based filtering step as done for the JF635-labeled samples above was applied, which removed spurious detections in autofluorescent blobs with a σ over 280 nm.

The fluorescence intensity of the JF646-BAPTA HTL depends on the local Ca^2+^ concentration, which changes with the channel’s activation state. Thus, the fluorescence intensity of the JF646-BAPTA HTL is higher when PIEZO1 channels are active/open than when in the resting/closed state; as channels flicker between open and closed, the fluorescence intensity of puncta varies over time (e.g. see kymograph in main Fig. 4F). Thus the time-based filtering used for the JF635 HTL above cannot be applied for image stacks of JF646-BAPTA HTL. Hence, Control JF 646-BAPTA PIEZO1-HaloTag KO and unlabeled PIEZO1-HaloTag MNRs were used to determine the threshold intensity values for separating puncta associated with active/open channels from unbound HTL and small autofluoresence puncta. Based on the distribution of integrated intensity of puncta in the Control samples, ~77 photons was selected as the filtering threshold. Puncta in JF646-BAPTA PIEZO1-HaloTag MNRs below this threshold were filtered out while puncta with intensities above this value were retained for analysis.

### Determining distance of PIEZO1-HaloTag puncta to MNR lumen and outer edge

In order to determine the distances between detected PIEZO1 puncta and the lumen or outer edge of the MNR, we manually drew masks identifying the lumen border and the outer edge of the rosettes on ImageJ, using the actin labeling as reference (example in main Figure 4D).

For the JF635-labeled PIEZO1-HaloTag experiments, we computed the mean position coordinates of each PIEZO1-HaloTag track. We then computed distances of this mean position for each track to the MNR lumen and outer edge masks using Euclidean distance transform, and rounded the distances up to the nearest pixel coordinates. The puncta overlapping with the masks were assigned a distance of zero for the corresponding mask. For the JF646-BAPTA-labeled PIEZO1-HaloTag signal, we used each filtered punctum localization as described above, and Euclidean distances from these to lumen and outer edges were similarly calculated.

The calculated distance distributions were visualized using density scatter plots. We used the “ks-density” function in MATLAB with a “normal” kernel having a bandwidth of [2.503 microns, 3.661 microns] for the distances to the lumen and outer edge respectively. Once the underlying distribution of the distances is obtained, the scatter plot was drawn using the “Scatter” object in MATLAB, with the colors corresponding to their probability densities. The transparency of the scatter points in the density scatter plots is based on the density values, where darker regions correspond to higher concentration of the puncta. This normalization method ensures similar smoothing and colormap scaling of the underlying distributions across all experiments, thus allowing us to compare distribution patterns across all the conditions tested.

The individual relative frequency distributions were plotted using histograms computed from the corresponding distance distributions with a bin size of 2 microns. For the histogram plots, the actual frequency of the detections at a particular distance from the corresponding mask was normalized using the total count, along with the bin size, so that the total area is 1. These plots were generated using the “hist” and “stairs” functions in MATLAB. Also, for further analysis, the respective cumulative distribution frequency (CDF) graphs were plotted using the “ecdf” function in MATLAB on the distance distributions, which computes the empirical CDF values from the data.

### 3D Detection and filtering of PIEZO1-HaloTag puncta in volumetric samples

The PIEZO1-HaloTag puncta localization in the volumetric data was inspected by numerically fitting a model of the PSF approximated by a 3D Gaussian function, as described previously^63^. JF635 PIEZO1-HaloTag KO MNRs were used as a control to determine filtering parameters to exclude autofluorescent blobs present in the data. For filtering, thresholding was applied on the fitted amplitude of the intensity above the local background, as well as the fitted local background to extract the detected diffraction limited puncta. This approach ensured that most puncta in the Knockout sample were removed, while retaining most of the puncta in JF635 PIEZO1-HaloTag MNRs.

## Supplementary Material

Supplement 1

Supplement 2

Supplement 3

Supplement 4

Supplement 5

Supplement 6

Supplement 7

Supplement 8

Supplement 9

Supplement 10
