# Supplementary material for: Visualizing PIEZO1 Localization and Activity in hiPSC-Derived Single Cells and Organoids with HaloTag Technology
Source: bioRxiv. 2025 Jan 25:2023.12.22.573117. Originally published 2023 Dec 23. Preprint. [Version 3] doi: 10.1101/2023.12.22.573117 (PMC10769387; doi:10.1101/2023.12.22.573117)
Supplement: Supplement 12 [file NIHPP2023.12.22.573117v3-supplement-12.pdf]

bioRxiv preprint doi: https://doi.org/10.1101/2023.12.22.573117; this version posted January 25, 2025. The copyright holder for this preprint (which was not certified by peer review) is the author/funder, who has granted bioRxiv a license to display the preprint in perpetuity. It is made available under aCC-BY 4.0 International license.

Supplementary Methods Information

Reagents

Table of Antibodies

Antibody	Host	Company, Cat. #	Use	Dilution
anti-PIEZO1	Mouse	Novus Biologicals, NBP2-75617	Western Blotting	1:1000
anti-HaloTag	Mouse	Promega, G9211	Western Blotting	1:1000
HRP-mouse anti actin	Mouse	ThermoFisher, MA5-15739-HRP	Western Blotting	1:100,000
anti-N-cadherin	Mouse	BD Biosciences, 610920	IF, Confocal	1:500
anti-Nestin	Mouse	R&D Systems, MAB1259	IF, Confocal	1:500
anti-Sox2	Rabbit	Millipore, AB5603	IF, confocal	1:500
anti-Sox2	Goat	R&D Systems, AF2018	IF, Confocal	1:300
anti-VE Cadherin	Rabbit	Abcam, ab33168	IF, Confocal	1:500
anti-CD31	Mouse	Agilent Technologies, M082329-2	IF, Confocal	1:500
anti-PAXILLIN	Mouse	Millipore, 05-41	IF, TIRF	1:200
anti-KERATIN 14	Rabbit	Biolegend, 905301	IF, TIRF	1:100
anti-Rabbit IgG	Goat	Invitrogen, A32731	IF	1:500
anti-Mouse IgG	Goat	Invitrogen, A32727	IF	1:500
anti-Goat IgG	Donkey	Invitrogen, A21432	IF	1:500

bioRxiv preprint doi: https://doi.org/10.1101/2023.12.22.573117; this version posted January 25, 2025. The copyright holder for this preprint (which was not certified by peer review) is the author/funder, who has granted bioRxiv a license to display the preprint in perpetuity. It is made available under aCC-BY 4.0 International license.

Table of HaloTag Ligands

HaloTag Ligand	Catalog No.	Concentration	Incubation Time
JF549	Cat. No. GA1110, Promega	500 pM	Overnight
JF635	Requested from Janelia Materials, Luke Lavis Lab	500 pM	Overnight
JF646	Cat. No. GA1120, Promega	500 pM	Overnight
JF646-BAPTA-3'AM	Requested from Janelia Materials, Luke Lavis Lab	500 pM	15 minutes

Chemical Reagents Table

Reagent	Catalog No.
mTeSR™ Plus Basal Medium	Cat. No.100-11300, STEMCELL Technologies
Primocin	Cat. No.NC9392943, Invivogen
Vitronectin XF	Cat. No.07180, STEMCELL Technologies
Accutase	Cat. No.# 07920_C, STEMCELL Technologies
Y-27632	Cat. No.SM-0013-0010, Biological Industries, USA
MatTek dishes	Cat. No.P35G-1.5-14-C, MatTek Corporation
MatTek dishes	Cat. No.P35G-1.5-20-C, MatTek Corporation
WTC-11	Coriell, GM4544
STEMdiff™ Neural Induction Medium	Cat. No. 05839, STEMCell Technologies
STEMdiff™ Neural Progenitor Medium	Cat. No. 05833, STEMCell Technologies
Advanced Dulbecco's modified Eagle's medium (DMEM)/F12	Cat. No. 12334010, Thermo Fisher Scientific
1x Glutamax supplement	Cat. No. 35050061, Thermo Fisher Scientific
L-Ascorbic Acid	Cat. No.A8960, Sigma- Aldrich
CHIR99021	SML1046-5MG, Sigma-Aldrich
SB431542	Cat. No. S1067, Selleck Chem
bFGF-2	Cat. No. 100-18B-100ug, PeproTech
Recombinant Human VEGF165 (VEGF-A)	Cat. No. 100-20, PeproTech
EGF	Cat. No. AF-100-15-100ug, PeproTech
CD31 MACs sorting kit	Cat. No. 130-091-935, Miltenyi
Fibronectin	Cat. No. 356008, Corning
EGM-2 media	Cat. No.CC-3162, Lonza
all-trans-RA	Cat. No. R2500-25MG, Sigma-Aldrich
Dispase	Cat. No.CnT-DNP-10, Cellntec
CnT-Prime Epithelial Proliferation Medium	Cat. No. CNT-PR, CellnTec

bioRxiv preprint doi: https://doi.org/10.1101/2023.12.22.573117; this version posted January 25, 2025. The copyright holder for this preprint (which was not certified by peer review) is the author/funder, who has granted bioRxiv a license to display the preprint in perpetuity. It is made available under aCC-BY 4.0 International license.

CnT-Prime Epithelial 2D Differentiation Medium	Cat. No. CnT-PR-D, CellnTec
CYTOOchips Arena A Chips	Cat. No.10-020-00-18, CYTOO INC.
CellAdhere™ Laminin-521	Cat. No.77003, STEMCELL Technologies
PBS with calcium and magnesium	Cat. No. PBL02-500ML, Caisson Laboratories Inc.
LDN 193189	Cat. No. SM-0005-0010, Biological Industries, USA
SPY555-actin	Cat. No. CY-SC202, Cytoskeleton
SPY505-DNA	Cat. No. CY-SC101, Cytoskeleton
35 mm dish without coverglass	Cellvis, cat. No. D35-14, Cellvis
KWIK-CAST	KWIK-CAST silicon sealant, World Precision Instruments
CD146 microbeads	Cat. No. 130-092-007, Miltenyi Biotech
DMEM/ F12 1:1	Cat. No. 25116001, Invitrogen
Standard HBSS	Cat. No. 14025092, GIBCO
FluoSpheres™ Carboxylate-Modified Microspheres (Crimson)	Cat. No. F8806, Invitrogen
FluoSpheres™ Carboxylate-Modified Microspheres (Yellow-Green)	Cat. No. F8811, Invitrogen

Table of Microscope information

**Microscope**	**Objective information (Magnification, Numerical Aperture)**	**Image Resolution Pixel Size (µm/pixel)**	**Filter Cubes Fluorescence Excitation/ Emission**
Keyence bZ-X810 Widefield Microscope	10x, NA 0.45<br>20x, NA 0.75<br>60x, NA 1.4 oil	1.50977<br>0.75488<br>0.12581	Ex 340-380/Em 435-485<br>Ex 465-495/Em 510 and up<br>Ex 527-553/Em 577-633<br>Ex 590-65/Em 662-737
Olympus FV3000S laser-scanning confocal microscope	40x, NA 1.25	0.3877	U-FUW Ex 365/50/Em 420 LP<br>U-FBN Ex 482/25/Em 510 LP<br>UFGW Ex 540/20/Em 575 LP
Olympus IX83 CellTIRF	60x, NA 1.45	0.1092	Ex FF01-563/9-25<br>Em FF01-624/40-25<br>Ex FF01-640/14-25<br>Em<br>FF01-676/29-25

bioRxiv preprint doi: https://doi.org/10.1101/2023.12.22.573117; this version posted January 25, 2025. The copyright holder for this preprint (which was not certified by peer review) is the author/funder, who has granted bioRxiv a license to display the preprint in perpetuity. It is made available under aCC-BY 4.0 International license.

Olympus IX83 CellTIRF	60x, NA 1.50	0.1082	Ex<br>ET-488/561nm Laser Dual TIRF Set for 486-492nm and 556-563nm lasers in BX3 cube<br>TRF49909-OL3; Single Bandpass ET-561nm laser TIRF set. For 557-563nm lasers. BX3 Cubes.<br>Em<br>E0431923; 455nm longpass cellTIRF Dichroic for 400-450nm lasers. Reflects: 400-450nm.<br>Transmits: 480-640nm.<br>E0431921; 525nm longpass cellTIRF Dichroic for 440-500nm lasers. Reflects: 440-500nm.<br>Transmits: 530-640nm.<br>E0431919; 595nm longpass cellTIRF Dichroic for 460-565nm lasers. Reflects: 460-565nm.<br>Transmits: 600-640nm.
AO-LLSM	20x, NA 1.0	0.108	CamA: Semrock FF01-600/ 37-25<br>CamB: Semrock FF01-538/685-25
