# Supplementary material for: PIEZO1-HaloTag hiPSCs: Bridging Molecular, Cellular and Tissue Imaging
Source: bioRxiv. 2023 Dec 23:2023.12.22.573117. Preprint. [Version 1] doi: 10.1101/2023.12.22.573117 (PMC10769387; doi:10.1101/2023.12.22.573117)
Supplement: Supplementary file 1 [file media-1.pdf]

Supplementary Information

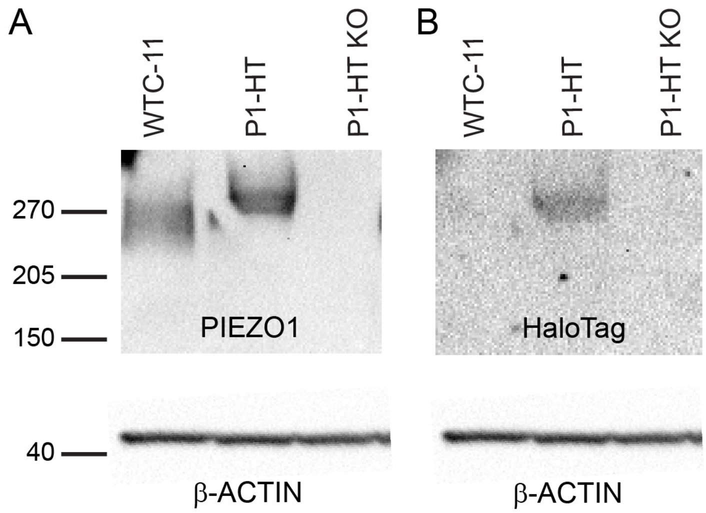

Supplemental Figure 1. Western blots demonstrate successful PIEZO1-HaloTag fusion. Immunoblot of hiPSCs (upper panels) probed with anti-PIEZO1 (**A)** or anti-HaloTag (**B)** antibody. The untagged PIEZO1 from WTC-11 hiPSCs (predicted molecular weight 289 kDa) and PIEZO1-HaloTag (predicted molecular weight 319 kDa) migrated at the expected sizes based on molecular weight standards. (Lower panels) Immunoblot probed with anti-β-ACTIN demonstrates equal loading of samples. n = 4 independent repeats.

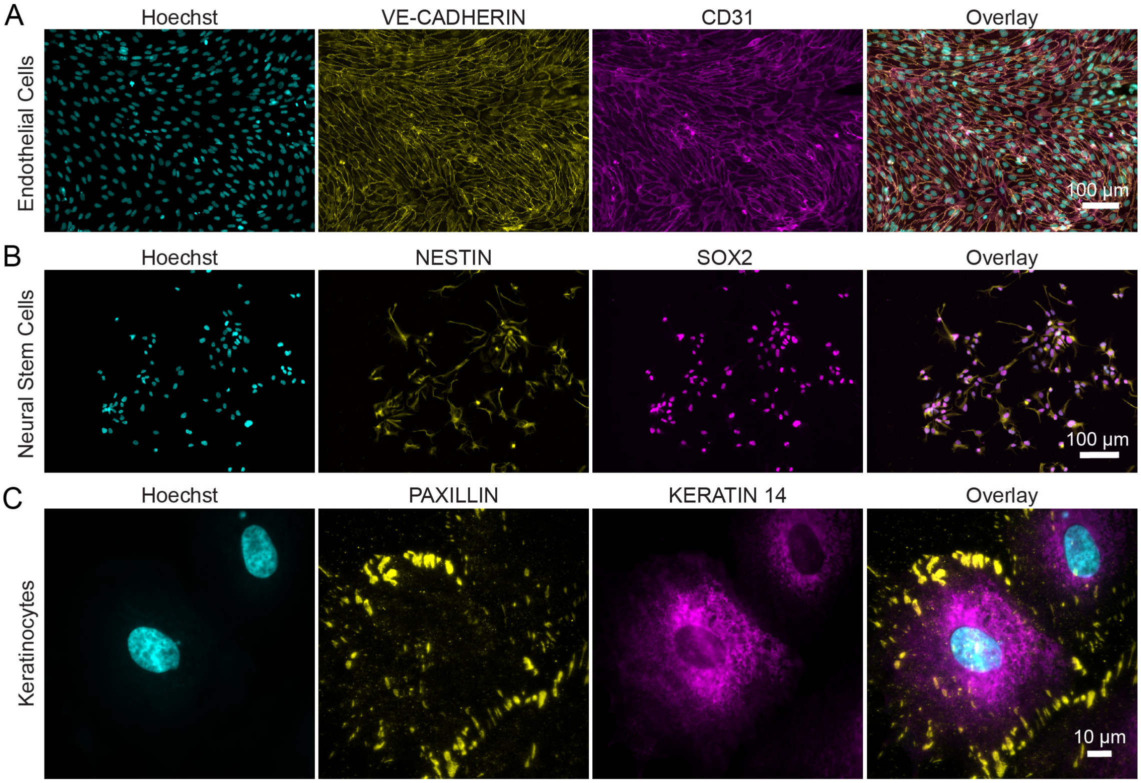

Supplemental Figure 2. Validation of endothelial cells, neural stem cells, and keratinocytes differentiated from PIEZO1-HaloTag hiPSCs. A. Endothelial cells differentiated from PIEZO1-HaloTag hiSPCs expressed endothelial cell markers VE-CADHERIN (Yellow) and CD31 (Magenta). **B.** Neural stem cells differentiated from PIEZO1-HaloTag hiPSCs expressed neural stem cell markers NESTIN (Yellow) and CD31 (Magenta). **C.** Keratinocytes differentiated from PIEZO1-HaloTag hiPSCs expressed keratinocyte marker KERATIN 14 (Magenta) and displayed prominent focal adhesion marker PAXILLIN (Yellow). See the Methods section for differentiation protocols.

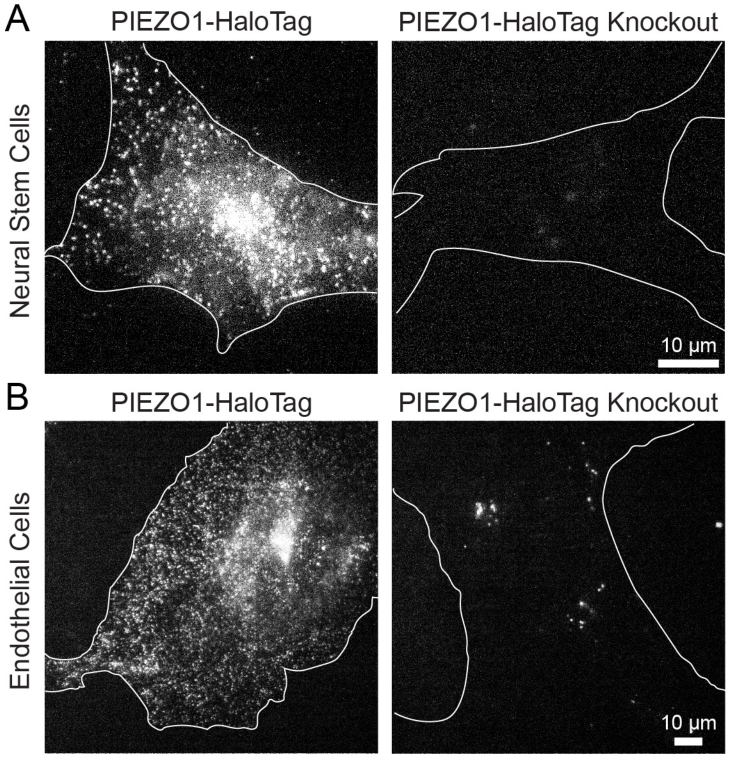

Supplemental Figure 3. Specific labeling of PIEZO1-HaloTag channels. A. Differentiated PIEZO1-HaloTag and PIEZO1-HaloTag Knockout neural stem cells labeled with JF646 HTL and imaged using TIRF. **B.** Differentiated PIEZO1-HaloTag and PIEZO1-HaloTag Knockout endothelial cells labeled with JF646 HTL and imaged using TIRF. Note the lack of HTL signal in Knockout cells.

Supp. Fig. 4

Untreated

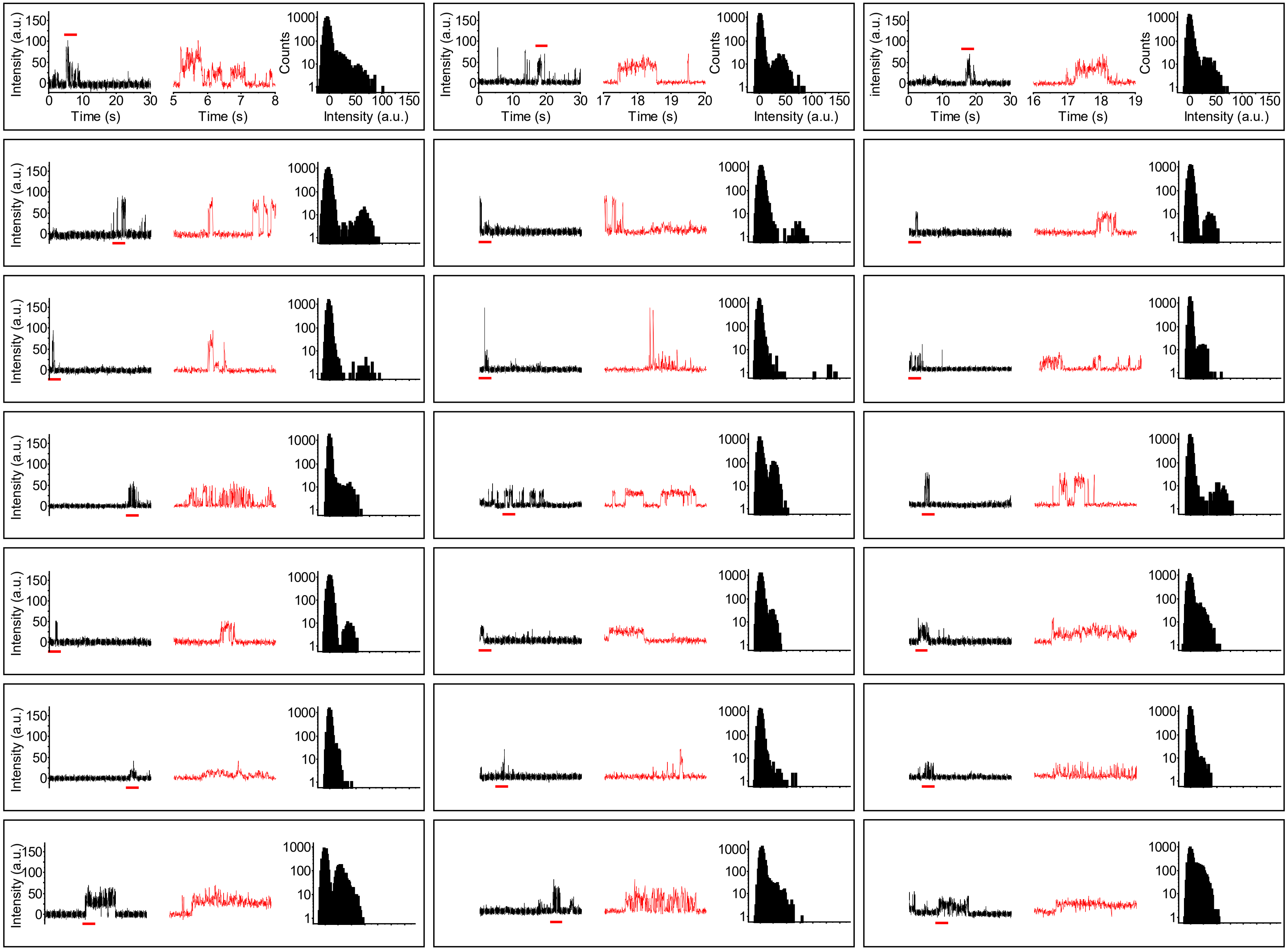

Supplemental Figure 4. Representative activity traces from untreated PIEZO1-HaloTag. Representative background-subtracted fluorescence intensity traces of 21 untreated immobile PIEZO1-HaloTag puncta from 3 independent TIRF imaging experiments of hiPSC-derived endothelial cells labeled with the Ca^{2+}-sensitive JF646-BAPTA HTL. Images were acquired at a frame rate of 200 fps. Intensity profiles were plotted from tracked immobile puncta. All traces shown are 30 seconds long (black) with a zoom in of a 3-second portion (red). Rightmost panels show an all-points histogram of intensity levels for the entirety of the 30-second recording.

Supp. Fig. 5

DMSO

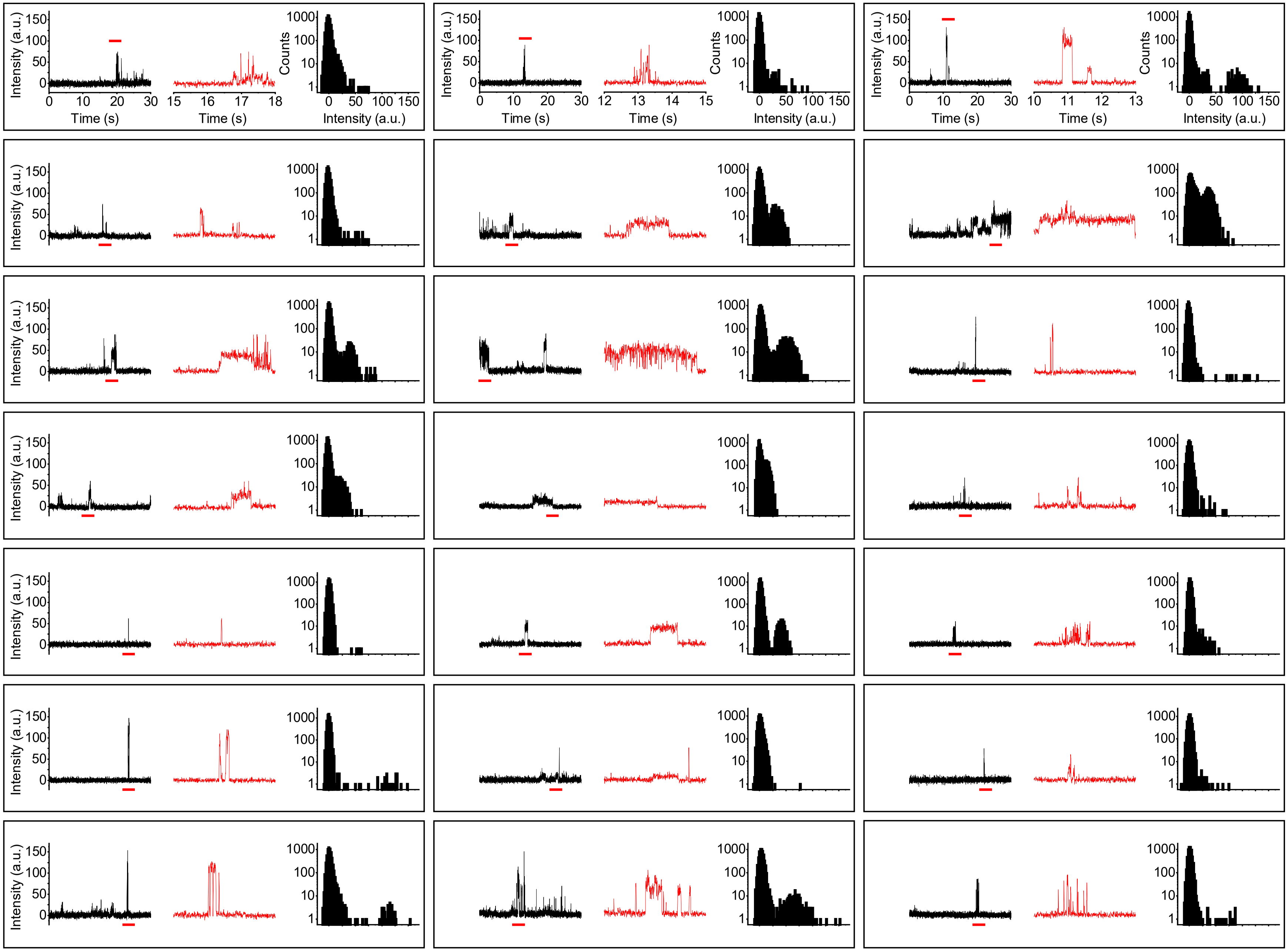

Supplemental Figure 5. Representative activity traces from PIEZO1-HaloTag treated with vehicle control, DMSO. Representative background-subtracted fluorescence intensity traces of 21 immobile PIEZO1-HaloTag puncta from 3 independent TIRF imaging experiments of hiPSC-derived endothelial cells labeled with the Ca^{2+}-sensitive JF646-BAPTA HTL and treated with vehicle control, DMSO. Intensity profiles were plotted from tracked immobile puncta. All traces shown are 30 seconds long (black) with a zoom in of a 3-second portion (red). Rightmost panels show an all-points histogram of intensity levels for the entirety of the 30-second recording.

Supp. Fig. 6

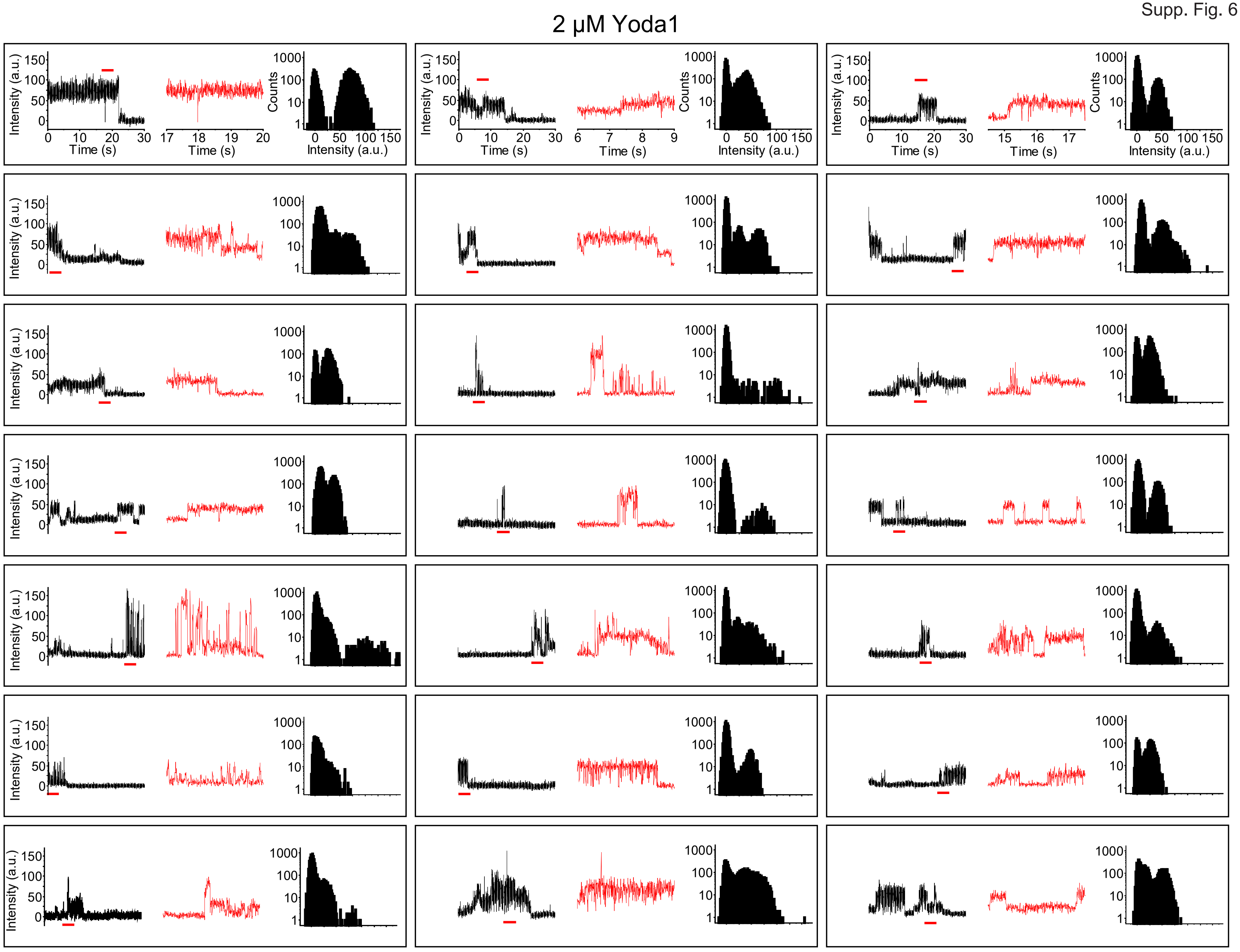

Supplemental Figure 6. Representative activity traces from PIEZO1-HaloTag treated with 2 μM of Yoda1. Representative background-subtracted fluorescence intensity traces of 21 immobile PIEZO1-HaloTag puncta from 3 independent TIRF imaging experiments of hiPSC-derived endothelial cells labeled with the Ca^{2+}-sensitive JF646-BAPTA HTL and treated with 2 μM Yoda1. Images were acquired at a frame rate of 200 fps. Intensity profiles were plotted from tracked immobile punctat. All traces shown are 30 seconds long (black) with a zoom in of a 3-second portion (red). Rightmost panels show an all-points histogram of intensity levels for the entirety of the 30-second recording.

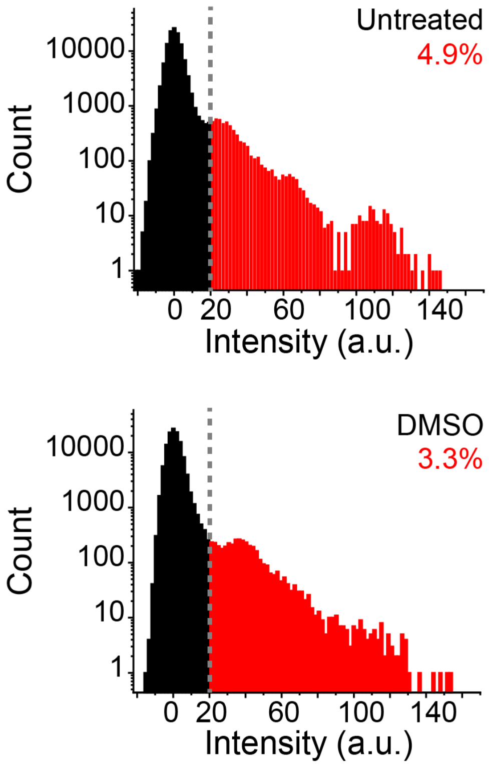

Supplemental Figure 7. PIEZO1-HaloTag Untreated and Vehicle Control open state occupancy. All-point histogram intensity levels from the traces in Supplemental Fig. 5 (Untreated) and Supplemental Fig. 6 (DMSO). Counts are shown on a log_{10} scale and are from 3 independent experiments. Counts above 20 au (red) represent open channels and are counted towards open state occupancy. Percentage numbers in red indicate the proportion of counts with fluorescence > 20 au. Counts below 20 au (black) are not incorporated into open state occupancy.

Density scatter plots of PIEZO1-HaloTag localizations

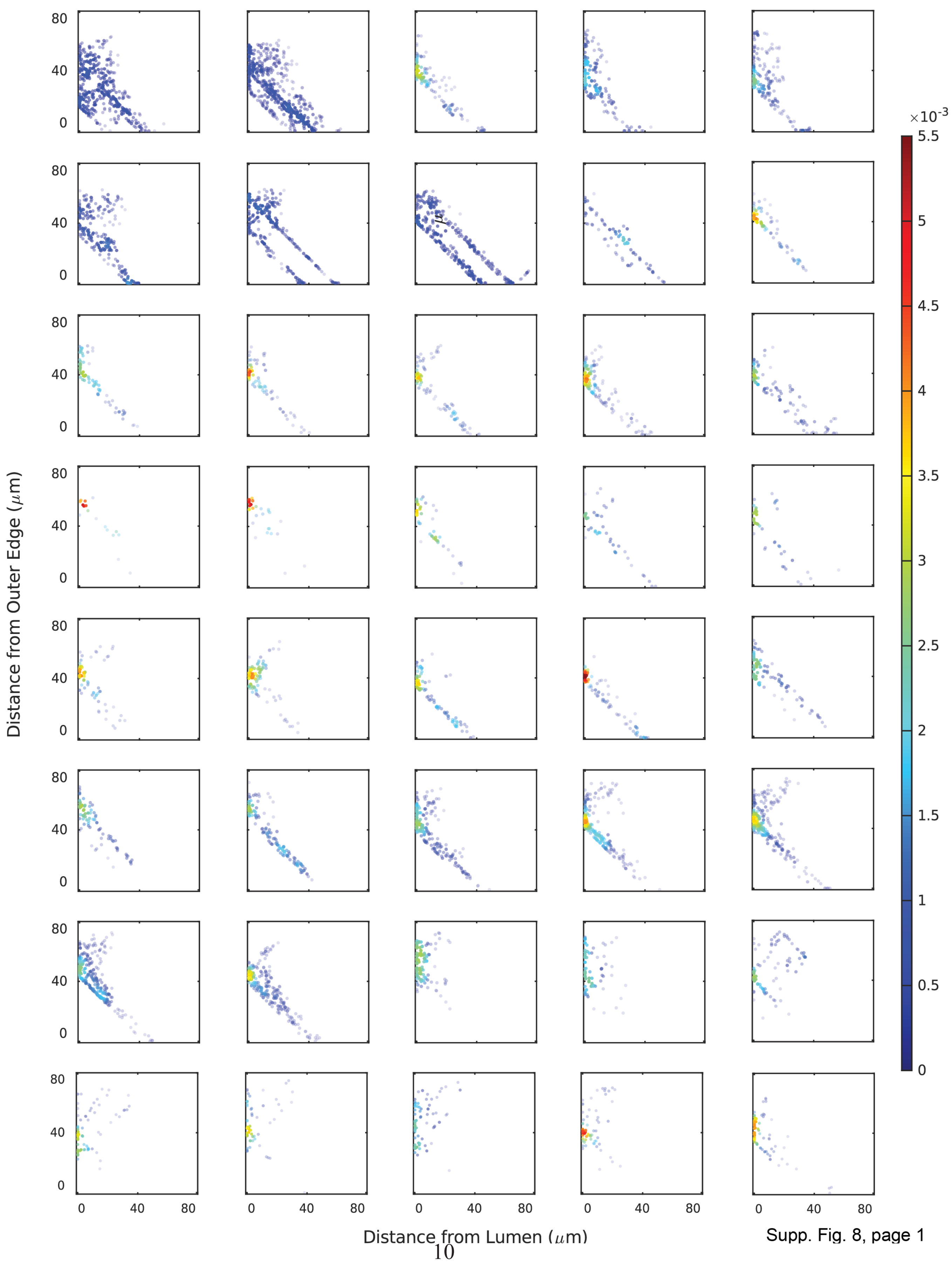

Supp. Fig. 8, page 1

Density scatter plots of PIEZO1-HaloTag localizations

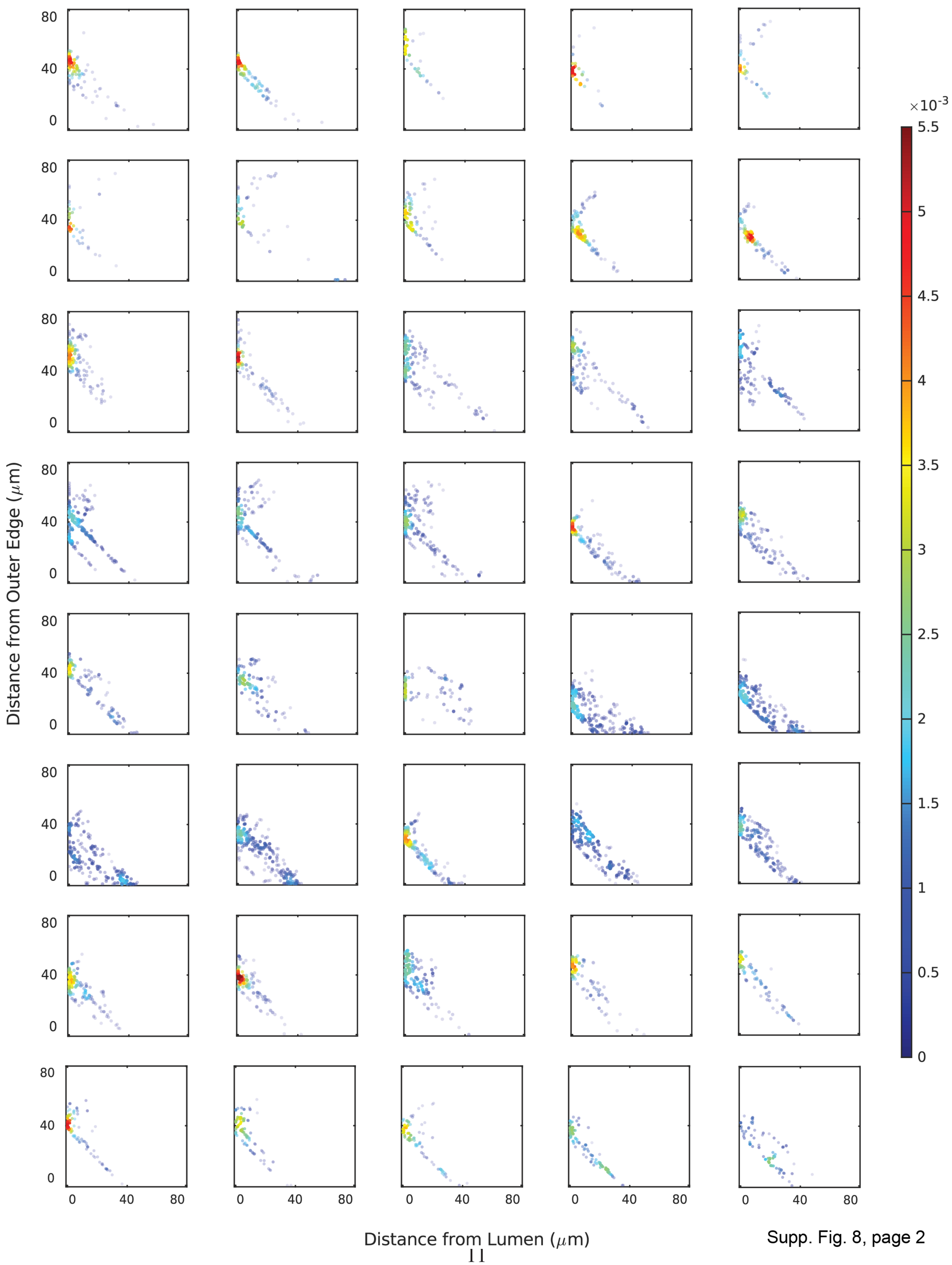

Supp. Fig. 8, page 2

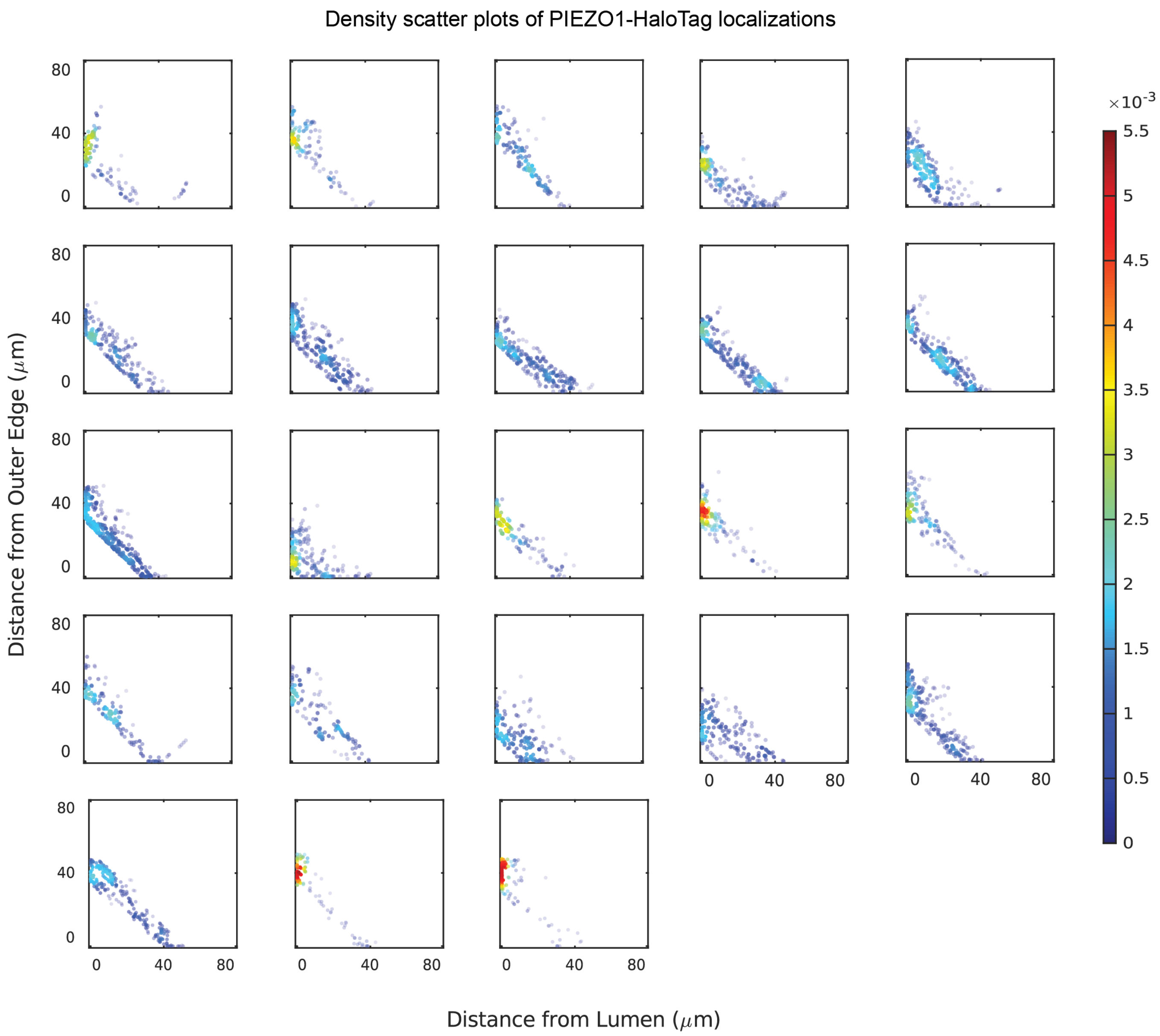

Supplemental Figure 8. Density scatter plots for individual MNRs labeled with JF635 HTL. The color scale indicates the relative density of puncta at each position in the scatter plot, normalized to the total number of puncta represented in the plot.

Supp. Fig. 8, page 3

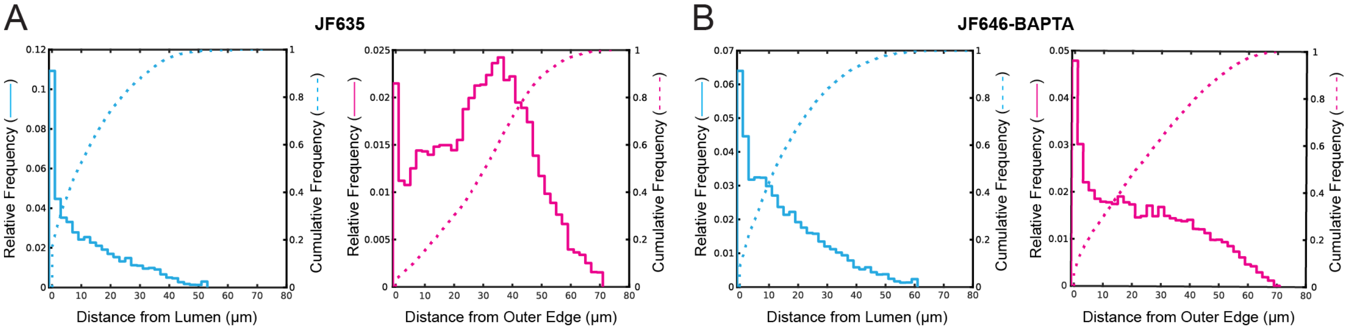

Supplemental Figure 9. Relative frequency plots for JF635 or JF646-BAPTA labeled MNRs. A. Relative frequency (solid lines) and cumulative frequency (dashed lines) of puncta distances to the lumen border (cyan) and outer edge (magenta) for JF635 labeled PIEZO1-HaloTag MNRs (n = 103 videos from 21 MNRs). **B.** The same as A, for JF646-BAPTA labeled MNRs (n = 39 videos from 12 MNRs). Cumulative frequency plots for each individual MNR can be found in Supplemental Fig. 10.

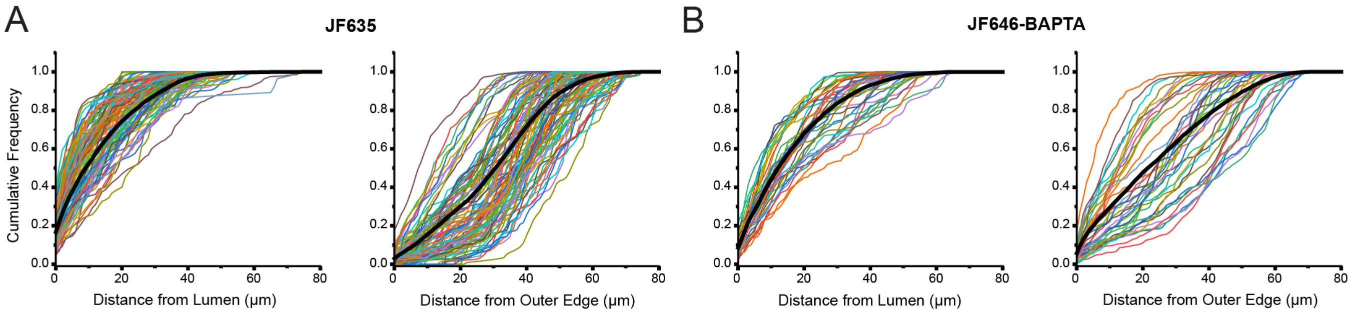

Supplemental Figure 10. Cumulative frequency plots for individual experiments of JF635 and JF646-BAPTA labeled MNRs. A. Left: Cumulative frequency plots showing distance of PIEZO1-HaloTag puncta from the MNR lumen border (left) or outer edge (right) for each individually imaged JF635 labeled MNR (n = 103 videos from 21 MNRs). Each color line denotes a different imaging video. The thick black line indicates the mean cumulative frequency for all experiments. **B.** As for A but for active PIEZO1-HaloTag puncta labeled with JF646-BAPTA (n = 39 videos from 12 MNRs).

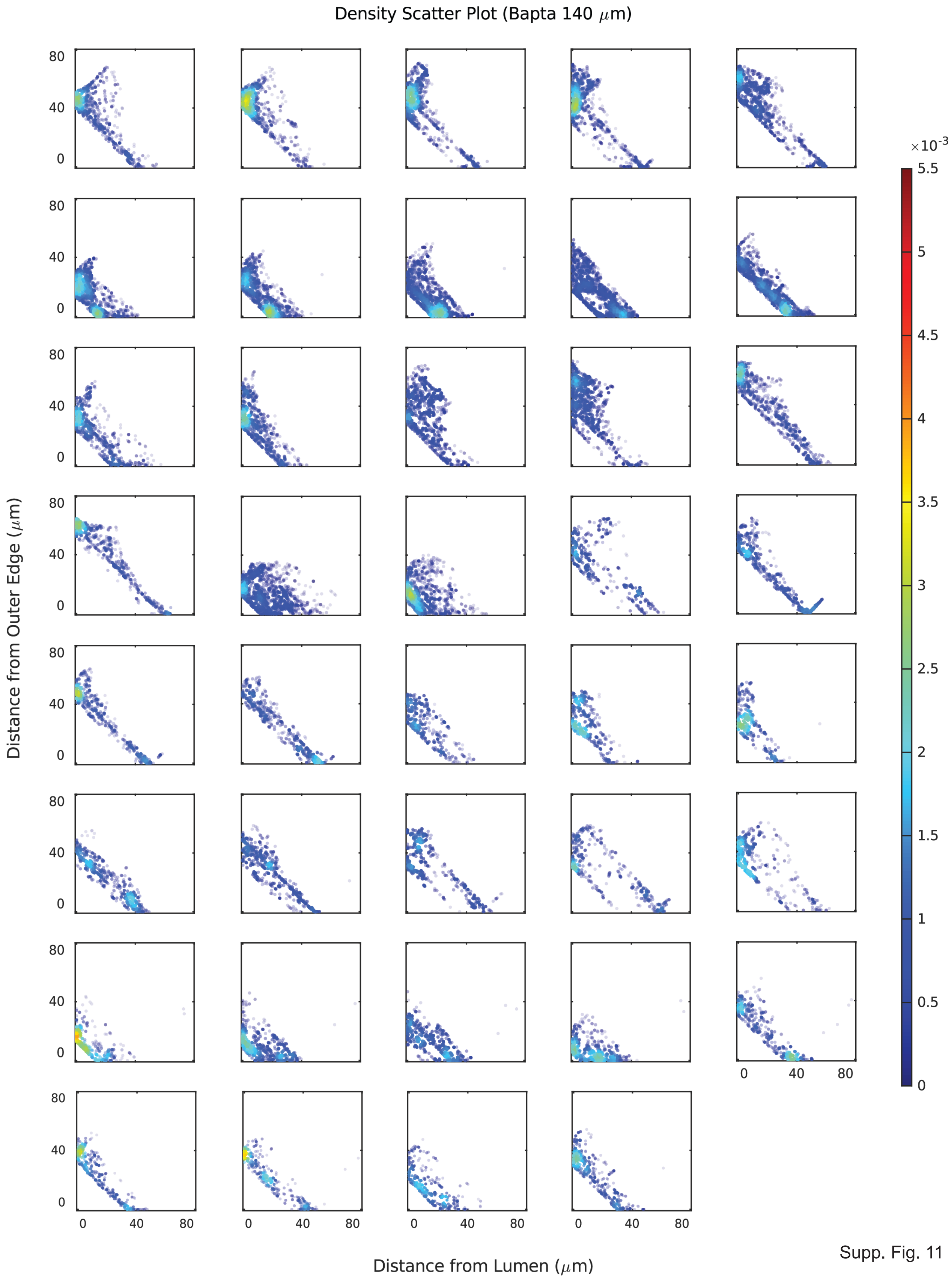

Density Scatter Plot (Bapta 140 μm)
Distance from Outer Edge (μm)
Distance from Lumen (μm)
×10-3

Supp. Fig. 11

Supplemental Figure 11. Density scatter plots for individual 140-µm MNRs labeled with JF646-BAPTA HTL. The color scale indicates the relative density of puncta at each position in the scatter plot, normalized to the total number of puncta represented in the plot.

Supplemental Videos

Supplemental Video 1. PIEZO1 puncta imaged in hiPSC-derived PIEZO1-HaloTag endothelial cells. A representative PIEZO1-HaloTag endothelial cell labeled with the JF646 HTL and imaged using TIRF microscopy for a duration of 2 minutes at 10 fps.

Supplemental Video 2. PIEZO1 puncta imaged in hiPSC-derived PIEZO1-HaloTag keratinocytes. A representative PIEZO1-HaloTag keratinocyte labeled with the JF646 HTL and imaged using TIRF microscopy for a duration of 2 minutes at 10 fps.

Supplemental Video 3. PIEZO1 puncta imaged in hiPSC-derived PIEZO1-HaloTag neural stem cells. A representative PIEZO1-HaloTag neural stem cell labeled with the JF646 HTL and imaged using TIRF microscopy for a duration of 2 minutes at 10 fps.

Supplemental Video 4. Localized enrichment of PIEZO1 at the rear of migrating NSC. A representative migrating PIEZO1-HaloTag neural stem cell labeled with the JF646 HTL and imaged using TIRF microscopy for 2 minutes at 10 fps.

Supplemental Video 5. Flickers from a trapped untreated PIEZO1-HaloTag JF646-BAPTA puncta. TIRF video of a trapped punctum from a PIEZO1-HaloTag endothelial cell (left). Green dot indicates centroid of trajectory and green box indicates 3x3 pixel region used to plot the fluorescence intensity trace populating on the right. Data acquired at a frame rate of 200 fps.

Supplemental Video 6. Flickers from a trapped DMSO treated PIEZO1-HaloTag JF646-BAPTA puncta. TIRF video of a trapped punctum from a PIEZO1-HaloTag endothelial cell(left) treated with vehicle control DMSO. Green dot indicates centroid of trajectory and green box indicates 3x3 pixel region used to generate the fluorescence intensity trace populating on the right. Data were acquired at a frame rate of 200 fps.

Supplemental Video 7. Flickers from a trapped 2 µM of Yoda1 treated PIEZO1-HaloTag JF646-BAPTA puncta.TIRF video a trapped punctum from a PIEZO1-HaloTag endothelial cell (left) treated with 2 µM of Yoda1. Green dot indicates centroid of trajectory and green box indicates 3x3 pixel region used to generate the fluorescence intensity trace populating on the right. Data were acquired at a frame rate of 200 fps.

Supplemental Video 8. Simultaneous imaging of PIEZO1-HaloTag JF646-BAPTA puncta mobility and activity. TIRF video of PIEZO1-HaloTag endothelial mobile puncta (left). Green dot indicates centroid of trajectory and the green line shows the trajectory of the puncta movement used to generate the fluorescence intensity trace populating on the right. Data were acquired at a frame rate of 200 fps.

Supplemental Video 9. 3D Visualization of actin and PIEZO1-HaloTag in MNR. PIEZO1-HaloTag in MNRs labeled with JF635 HTL imaged using AO-LLSM and visualized using both 2D orthoslices and 3D volumetric views. The video shows actin (white), denoised nuclei (cyan), PIEZO1-HaloTag (green, raw data; magenta, computationally detected PIEZO1 puncta), followed by a 3D local density map of the PIEZO1-HaloTag detections color-coded for the total number of detected PIEZO1-HaloTag puncta within a 3.6µm search radius or 200μm^3 search volume centered around each detected PIEZO1 puncta.

Supplementary Results

A size- and time-based detection filtering method removed non-specific puncta detections in JF635-labeled MNRs

Lightsheet imaging of the PIEZO1-HaloTag in micropatterned neural rosettes (MNRs) allowed us to study PIEZO1 localization and activity in a model of human neural development. Specifically, we used this system to determine the distance between PIEZO1-HaloTag puncta and the lumen border or the outer edge of the MNR (see main Figure 4D-G). To study puncta localization, we first used the ThunderSTORM detection algorithm to detect puncta in JF635-labeled PIEZO1-HaloTag MNRs; JF635-labeled PIEZO1-HaloTag KO MNRs as well as unlabeled PIEZO1-HaloTag MNRs served as controls (see Methods). Analysis of PIEZO1-HaloTag MNRs displayed 91% more detections per MNR than PIEZO1-HaloTag KO MNRs (percentage calculated from the median values of each condition, see Supplemental Fig. 12A). However, some of the detected puncta were attributed to non-specific autofluorescent blobs and to unbound HTL molecules, as these were also present in the two control samples. Therefore, we developed a method to computationally filter out these spurious detections and enrich the pool of detections representing bona fide PIEZO1-HaloTag puncta.

Most of the non-specific detections appeared within large autofluorescence blobs also present in control KO and unlabeled MNRs (Supplemental Fig. 12B-D). These spots were immobile, presented stable intensity profiles over time (except for a progressive intensity decrease due to bleaching), and were much larger than PIEZO1-HaloTag puncta. Therefore, we applied a first filtering step based on the size of puncta, in which we removed any initial detections that had a standard deviation (**σ**) of their Gaussian fit larger than 280 nm. This step successfully removed most detections that were located within autofluorescence blobs (Supplemental Fig. 12B-D).

Non-specific spurious detections corresponding to unbound JF635 HTL were visible in JF635-labeled PIEZO1-HaloTag KO MNRs (Supplemental Fig. 12B-D). These detections generally appeared for only one or two frames, while bona fide PIEZO1-HaloTag puncta observed in JF635 PIEZO1-HaloTag MNRs tended to last several frames (Supplemental Fig. 12E). We thus used the FLIKA algorithm to track puncta over time and then applied a second, time-based filtering step designed to remove spurious detections that lasted only for one or two frames of imaging (Supplemental Fig. 12B-D).

After these two filtering steps, JF635 PIEZO1-HaloTag MNRs contained 9.6 times more puncta than JF635 PIEZO1-HaloTag KO MNRs, and 6.9 times more than unlabeled PIEZO1-HaloTag MNRs (Supplemental Fig. 12F). Therefore, our puncta filtering method allowed us to remove detections that were not representative of bona fide PIEZO1 puncta, and enrich the proportion of detections representative of PIEZO1 puncta in control samples for further analysis.

A size- and intensity-based detection filtering method removed non-specific puncta detections in JF646-BAPTA-labeled NMRs

Light-sheet imaging of MNRs labeled with JF646-BAPTA HTL also enabled us to quantify the spatial distribution of active puncta in the neural organoids. Before analyzing the data, we conducted two filtering steps to remove nonspecific detections. The first step was identical to the size-based step used for JF635 samples above, while the second step was tailored to the highly variable intensity profiles displayed by BAPTA HTL ligands.

Upon initial puncta detection with ThunderSTORM, JF646-BAPTA PIEZO1-HaloTag MNRs presented 59% more detections than JF646-BAPTA PIEZO1-HaloTag KO MNRs (Supplemental Fig. 13A). We first filtered out any puncta with a **σ** over 280 nm, which removed detections in large autofluorescence blobs (Supplemental Fig. 13B-D).

Because BAPTA probes flicker quickly over time (see kymograph in main Fig. 4F), the second filtering step was based on puncta intensity rather than track duration (Supplemental Fig. 13B-D). Here we removed puncta with a value of integrated intensity lower than ~77 photons, based on the distribution of integrated intensity values in control samples. After filtering, JF646-BAPTA PIEZO1-HaloTag MNRs had 2.6 times more localizations than JF646-BAPTA PIEZO1-HaloTag KO MNRs, and 2 times more than unlabeled PIEZO1-HaloTag MNRs (Supplemental Fig. 13D). Only detections that were both small enough in spatial spread and bright enough to represent active PIEZO1 puncta were then retained for analysis of active punta localization in MNRs. Therefore, our method successfully enriched the initial pool of detections in JF646-BAPTA labeled MNRs with bona fide active PIEZO1 puncta.

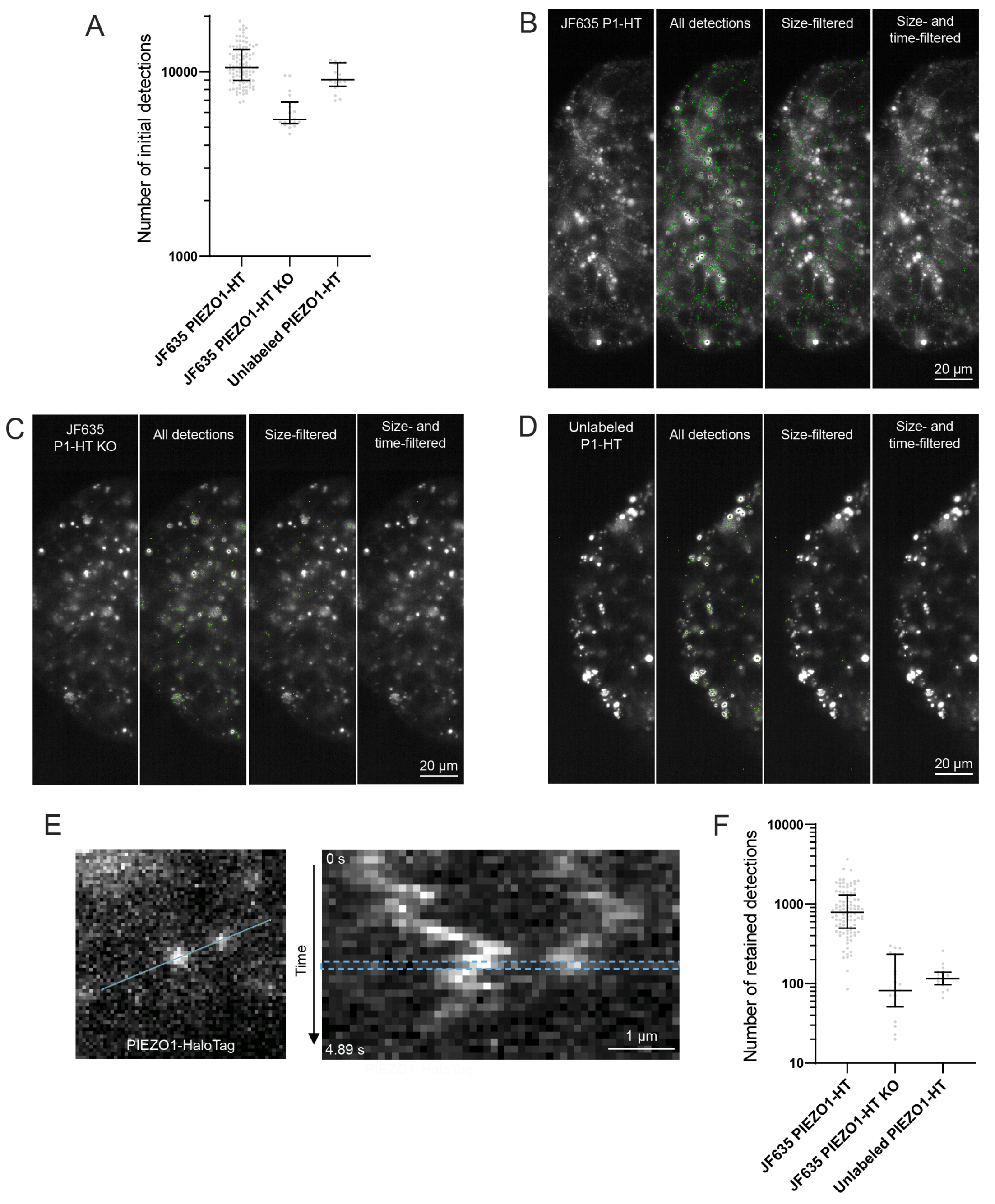

Supplemental Figure 12. Size- and time-based filtering removes non-specific localizations in JF635-labeled MNRs. A. Number of puncta detected by ThunderSTORM in each sample type. Median values: JF635 PIEZO1-HT: 10579 detections (n=103 videos from 21 MNRs), JF635 PIEZO1-HT KO: 5528 detections (n=19 videos from 6 MNRs), Unlabeled PIEZO1-HT: 9046 detections (n=16 videos from 2 MNRs). **B.** Representative images of puncta detections remaining after each step in the puncta filtering process in the first data frame of a JF635 PIEZO1-HaloTag MNR. From left to right: PIEZO1-HaloTag data, initial detected puncta, remaining puncta after size-based filtering, and remaining puncta after time-based filtering. **C.** As in B, but for a JF635 PIEZO1-HaloTag Knockout MNR. **D.** As in B and C, but for an unlabeled PIEZO1-HaloTag MNR. **E.** Representative 3-plane stack image of JF635 PIEZO1-HaloTag puncta in an MNR. The blue line indicates the region of interest used to generate the kymograph on the right. The dashed blue rectangle in the kymograph indicates the frame shown in the left panel. Note how JF635 PIEZO1-HaloTag puncta remain bright over several frames, in contrast to similar kymograph shown in Fig. 4F for JF646-BAPTA HTL which displays flickering. **F.** Number of remaining puncta in each sample type after size- and time-based filtering. The two-step filtering process resulted in a substantial reduction of detections in JF635 PIEZO1-HT KO MNRs and unlabeled PIEZO1-HaloTag MNRs, with a smaller reduction of puncta in JF635 PIEZO1-HaloTag MNRs. Median values: JF635 PIEZO1-HT: 788 detections (n=103 videos from 21 MNRs), JF635 PIEZO1-HT KO: 82 detections (n=19 videos from 6 MNRs), Unlabeled PIEZO1-HT: 115 detections (n=16 videos from 2 MNRs). **All plots:** Each dot is one video, bars are the median with interquartile range for each condition.

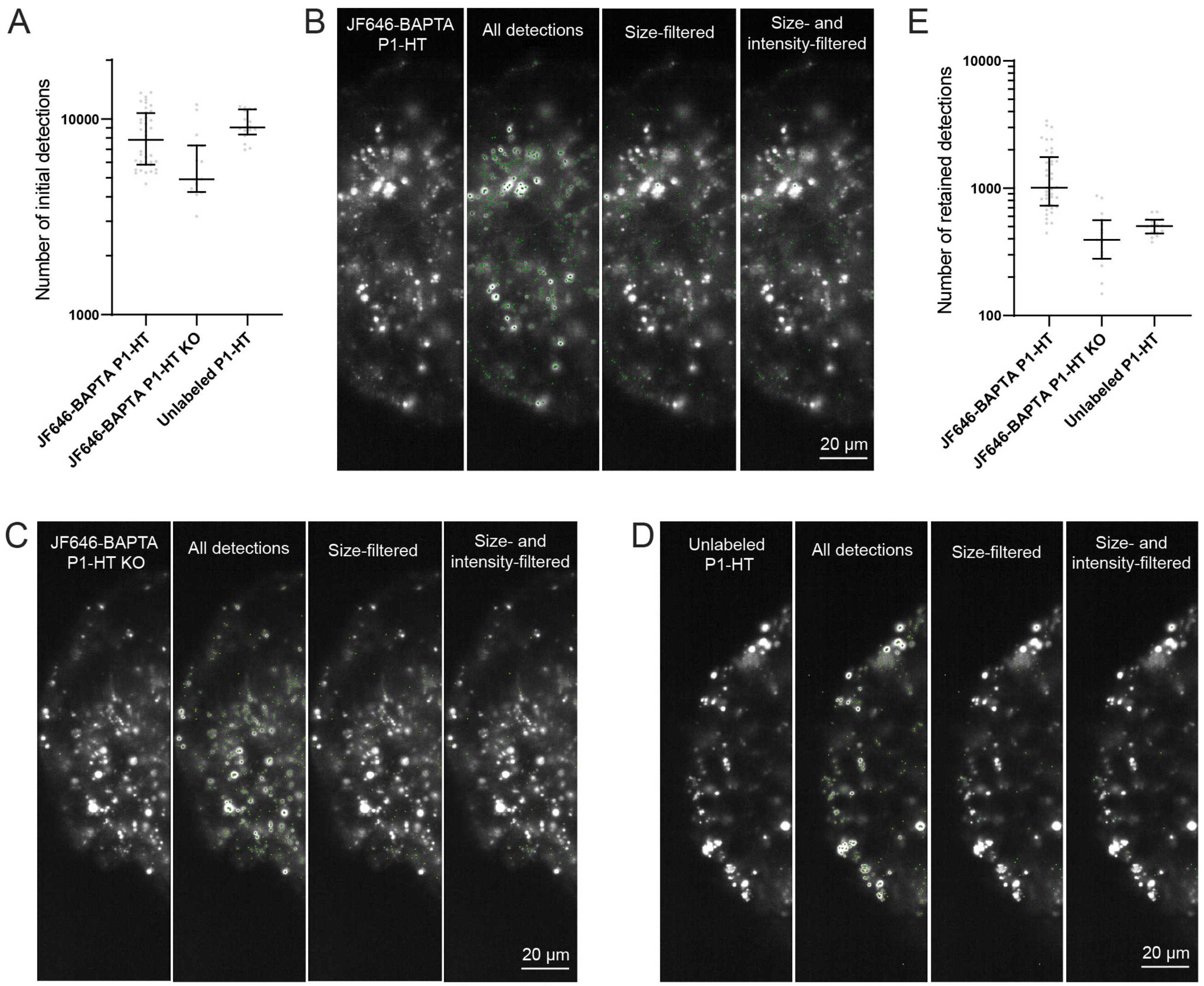

Supplemental Figure 13. Size- and intensity-based filtering removes non-specific localizations in JF646-BAPTA-labeled MNRs. **A.** Number of puncta initially detected by ThunderSTORM in each sample type. Median values: JF646-BAPTA PIEZO1-HT: 7834 detections (n=39 videos from 12 MNRs), JF646-BAPTA PIEZO1-HT KO: 4928 detections (n=15 videos from 3 MNRs), Unlabeled PIEZO1-HT: 9046 detections (n=16 videos from 2 MNRs). **B.** Representative images of puncta detections remaining after each step in the puncta filtering process in the first data frame of a JF646-BAPTA PIEZO1-HaloTag MNR. From left to right: PIEZO1-HaloTag data, initially detected puncta, remaining puncta after size-based filtering, and remaining puncta after intensity-based filtering. **C.** As in B, but for a JF646-BAPTA PIEZO1-HaloTag Knockout MNR. **D.** As in B and C, but for an unlabeled PIEZO1-HaloTag MNR. **E.** Number of remaining puncta in each sample type after size- and intensity-based filtering. The two-step filtering process resulted in a substantial reduction of detections in JF646-BAPTA PIEZO1-HT KO MNRs and unlabeled PIEZO1-HaloTag MNRs, with a smaller reduction of puncta in JF646-BAPTA PIEZO1-HaloTag MNRs. Median values: JF635 PIEZO1-HT: 1009 detections (n=39 videos from 12 MNRs), JF635 PIEZO1-HT KO: 393 detections (n=15 videos from 3 MNRs), Unlabeled PIEZO1-HT: 504 detections (n=16 videos from 2 MNRs). **All plots:** Each dot is one time series, bars are the median with interquartile range.

upplementary Methods Information:

Table of Antibodies

Antibody	Host	Company, Cat. #	Use	Dilution
anti-N-cadherin	Mouse	BD Biosciences, 610920	IF, Confocal	1:500
anti-Nestin	Mouse	R&D Systems, MAB1259	IF, Confocal	1:500
anti-Sox2	Rabbit	Millipore, AB5603	IF, confocal	1:500
anti-Sox2	Goat	R&D Systems, AF2018	IF, Confocal	1:300
anti-VE Cadherin	Rabbit	Abcam, ab33168	IF, Confocal	1:500
anti-CD31	Mouse	Agilent Technologies, M082329-2	IF, Confocal	1:500
anti-PAXILLIN	Mouse	Millipore, 05-41	IF, TIRF	1:200
anti-KERATIN 14	Rabbit	Biolegend, 905301	IF, TIRF	1:100
anti-Rabbit IgG	Goat	Invitrogen, A32731	IF	1:500
anti-Mouse IgG	Goat	Invitrogen, A32727	IF	1:500
anti-Goat IgG	Donkey	Invitrogen, A21432	IF	1:500

Table of HaloTag Ligands

HaloTag Ligand	Catalog No.
JF549	Cat. No. GA1110, Promega
JF635	Requested from Janelia Materials, Luke Lavis Lab
JF646	Cat. No. GA1120, Promega
JF646-BAPTA-3'AM	Requested from Janelia Materials, Luke Lavis Lab

Chemical Reagents Table

Reagent	Catalog No.
mTeSR™ Plus Basal Medium	Cat. No.100-11300, STEMCELL Technologies
Primocin	Cat. No.NC9392943, Invivogen
Vitronectin XF	Cat. No.07180, STEMCELL Technologies
Accutase	Cat. No.# 07920_C, STEMCELL Technologies
Y-27632	Cat. No.SM-0013-0010, Biological Industries, USA
MatTek dishes	Cat. No.P35G-1.5-14-C, MatTek Corporation
MatTek dishes	Cat. No.P35G-1.5-20-C, MatTek Corporation
WTC-11	Coriell, GM4544
STEMdiff™ Neural Induction Medium	Cat. No. 05839, STEMCell Technologies
Advanced Dulbecco's modified Eagle's medium (DMEM)/F12	Cat. No. 12334010, Thermo Fisher Scientific
1x Glutamax supplement	Cat. No. 35050061, Thermo Fisher Scientific

L-Ascorbic Acid	Cat. No.A8960, Sigma- Aldrich
CHIR99021	SML1046-5MG, Sigma-Aldrich
SB431542	Cat. No. S1067, Selleck Chem
bFGF-2	Cat. No. 100-18B-100ug, PeproTech
Recombinant Human VEGF165 (VEGF-A)	Cat. No. 100-20, PeproTech
EGF	Cat. No. AF-100-15-100ug, PeproTech
CD31 MACs sorting kit	Cat. No. 130-091-935, Miltenyi
Fibronectin	Cat. No. 356008, Corning
EGM-2 media	Cat. No.CC-3162, Lonza
all-trans-RA	Cat. No. R2500-25MG, Sigma-Aldrich
Dispase	Cat. No.CnT-DNP-10, Cellntec
CnT-Prime Epithelial Proliferation Medium	Cat. No. CNT-PR, CellnTec
CnT-Prime Epithelial 2D Differentiation Medium	Cat. No. CnT-PR-D, CellnTec
CYTOOchips Arena A Chips	Cat. No.10-020-00-18, CYTOO INC.
CellAdhere™ Laminin-521	Cat. No.77003, STEMCELL Technologies
PBS with calcium and magnesium	Cat. No. PBL02-500ML, Caisson Laboratories Inc.
LDN 193189	Cat. No. SM-0005-0010, Biological Industries, USA
SPY555-actin	Cat. No. CY-SC202, Cytoskeleton
SPY505-DNA	Cat. No. CY-SC101, Cytoskeleton
35 mm dish without coverglass	Cellvis, cat. No. D35-14, Cellvis
KWIK-CAST	KWIK-CAST silicon sealant, World Precision Instruments
CD146 microbeads	Cat. No. 130-092-007, Miltenyi Biotech
DMEM/ F12 1:1	Cat. No. 25116001, Invitrogen

Table of Microscope information

**Microscope**	**Objective information (Magnification, Numerical Aperture)**	**Image Resolution Pixel Size (µm/pixel)**	**Filter Cubes Fluorescence Excitation/ Emission**
Keyence bZ-X810 Wide-field Microscope	10x, NA 0.45<br>20x, NA 0.75<br>60x, NA 1.4 oil	1.50977<br>0.75488<br>0.12581	Ex 340-380/Em 435-485<br>Ex 465-495/Em 510 and up<br>Ex 527-553/Em 577-633<br>Ex 590-65/Em 662-737

Olympus FV3000S laser-scanning confocal microscope	40x, NA 1.25	0.3877	U-FUW Ex 365/50/Em 420 LP<br>U-FBN Ex 482/25/Em 510 LP<br>UFGW Ex 540/20/Em 575 LP
Olympus IX83 CellTIRF	60x, NA 1.45	0.1092	Ex FF01-563/9-25<br>Em FF01-624/40-25<br>Ex FF01-640/14-25<br>Em<br>FF01-676/29-25
Olympus IX83 CellTIRF	60x, NA 1.50	0.1082	Ex<br>ET-488/561nm Laser Dual TIRF Set for 486-492nm and 556-563nm lasers in BX3 cube<br>TRF49909-OL3; Single Bandpass ET-561nm laser TIRF set. For 557-563nm lasers. BX3 Cubes.<br>Em<br>E0431923; 455nm longpass cellTIRF Dichroic for 400-450nm lasers. Reflects: 400-450nm.<br>Transmits: 480-640nm.<br>E0431921; 525nm longpass cellTIRF Dichroic for 440-500nm lasers. Reflects: 440-500nm.<br>Transmits: 530-640nm.<br>E0431919; 595nm longpass cellTIRF Dichroic for 460-565nm lasers. Reflects: 460-565nm.<br>Transmits: 600-640nm.
AO-LLSM	20x, NA 1.0	0.108	CamA: Semrock FF01-600/ 37-25<br>CamB: Semrock FF01-538/685-25

Software used

OriginPro 2022 (64-bit) SR1 9.9.0.225

FLIKA version 4.8.1

ImageJ 2.9.0/1.53t ; Java 1.8.0_322

Micro-Manager 2.0

cellTIRF 1.4

Python 3.11.5

Imaris x64: 10.0.0

Amira 3D 2021.1

MATLAB versions R2022b and R2023a
